# Mutually exclusive substrate selection strategy by human m^3^C RNA transferases METTL2A and METTL6

**DOI:** 10.1093/nar/gkab603

**Published:** 2021-07-15

**Authors:** Xue-Ling Mao, Zi-Han Li, Meng-Han Huang, Jin-Tao Wang, Jing-Bo Zhou, Qing-Run Li, Hong Xu, Xi-Jin Wang, Xiao-Long Zhou

**Affiliations:** State Key Laboratory of Molecular Biology, CAS Center for Excellence in Molecular Cell Science, Shanghai Institute of Biochemistry and Cell Biology, Chinese Academy of Sciences, University of Chinese Academy of Sciences, 320 Yue Yang Road, Shanghai 200031, China; State Key Laboratory of Molecular Biology, CAS Center for Excellence in Molecular Cell Science, Shanghai Institute of Biochemistry and Cell Biology, Chinese Academy of Sciences, University of Chinese Academy of Sciences, 320 Yue Yang Road, Shanghai 200031, China; State Key Laboratory of Molecular Biology, CAS Center for Excellence in Molecular Cell Science, Shanghai Institute of Biochemistry and Cell Biology, Chinese Academy of Sciences, University of Chinese Academy of Sciences, 320 Yue Yang Road, Shanghai 200031, China; State Key Laboratory of Molecular Biology, CAS Center for Excellence in Molecular Cell Science, Shanghai Institute of Biochemistry and Cell Biology, Chinese Academy of Sciences, University of Chinese Academy of Sciences, 320 Yue Yang Road, Shanghai 200031, China; State Key Laboratory of Molecular Biology, CAS Center for Excellence in Molecular Cell Science, Shanghai Institute of Biochemistry and Cell Biology, Chinese Academy of Sciences, University of Chinese Academy of Sciences, 320 Yue Yang Road, Shanghai 200031, China; CAS Key Laboratory of Systems Biology, CAS Center for Excellence in Molecular Cell Science, Shanghai Institute of Biochemistry and Cell Biology, Chinese Academy of Sciences, University of Chinese Academy of Sciences, 320 Yue Yang Road, Shanghai 200031, China; Shanghai Key Laboratory of Embryo Original Diseases, the International Peace Maternity and Child Health Hospital, School of Medicine, Shanghai Jiao Tong University, 910 Heng Shan Road, Shanghai 200030, China; Department of Neurology, Xinhua Hospital, School of Medicine, Shanghai Jiao Tong University, 1665 Kong Jiang Road, Shanghai 200092, China; State Key Laboratory of Molecular Biology, CAS Center for Excellence in Molecular Cell Science, Shanghai Institute of Biochemistry and Cell Biology, Chinese Academy of Sciences, University of Chinese Academy of Sciences, 320 Yue Yang Road, Shanghai 200031, China

## Abstract

tRNAs harbor the most diverse posttranscriptional modifications. The 3-methylcytidine (m^3^C) is widely distributed at position C32 (m^3^C32) of eukaryotic tRNA^Thr^ and tRNA^Ser^ species. m^3^C32 is decorated by the single methyltransferase Trm140 in budding yeasts; however, two (Trm140 and Trm141 in fission yeasts) or three enzymes (METTL2A, METTL2B and METTL6 in mammals) are involved in its biogenesis. The rationale for the existence of multiple m^3^C32 methyltransferases and their substrate discrimination mechanism is hitherto unknown. Here, we revealed that both METTL2A and METTL2B are expressed *in vivo*. We purified human METTL2A, METTL2B, and METTL6 to high homogeneity. We successfully reconstituted m^3^C32 modification activity for tRNA^Thr^ by METT2A and for tRNA^Ser^(GCU) by METTL6, assisted by seryl-tRNA synthetase (SerRS) *in vitro*. Compared with METTL2A, METTL2B exhibited dramatically lower activity *in vitro*. Both G35 and t^6^A at position 37 (t^6^A37) are necessary but insufficient prerequisites for tRNA^Thr^ m^3^C32 formation, while the anticodon loop and the long variable arm, but not t^6^A37, are key determinants for tRNA^Ser^(GCU) m^3^C32 biogenesis, likely being recognized synergistically by METTL6 and SerRS, respectively. Finally, we proposed a mutually exclusive substrate selection model to ensure correct discrimination among multiple tRNAs by multiple m^3^C32 methyltransferases.

## INTRODUCTION

Transfer RNA (tRNA) is the most highly and diversely modified RNA species in the cell (1). To date, among 143 currently known modified ribonucleosides, 111 modifications have been identified in tRNAs from all three domains of life (2). tRNA modifications frequently occur in loop regions, such as the D-loop, TψC-loop and anticodon loop, to maintain stability of the tRNA architecture and/or guarantee fidelity and efficiency during ribosomal translation at the decoding site, thereby regulating gene expression and protein homeostasis (3,4).

3-Methylcytidine (m^3^C) (Supplementary Figure S1A) modification is widely found in eukaryotic cytoplasmic tRNA^Thr^, tRNA^Ser^, a subset of tRNA^Arg^ species, and mammalian mitochondrial tRNA^Thr^ and tRNA^Ser^(UCN) at position 32 of the anticodon loop. In addition, it is present at base 20 of mammalian elongator tRNA^Met^ [tRNA^Met^(e)] and at base 47d (e2) of mammalian tRNA^Leu^(CAG) and all tRNA^Ser^ species (Supplementary Figure S1B) (5). Only the methyltransferases catalyzing m^3^C at position 32 (m^3^C32) of eukaryotic cytoplasmic tRNA^Thr^, tRNA^Ser^ and tRNA^Arg^ have been identified (Supplementary Figure S1B) (Supplementary Table S1) (6–8). In the budding yeast *Saccharomyces cerevisiae*, only a single enzyme, Trm140 (*Sc*Trm140), introduces m^3^C32 in both tRNA^Thr^ and tRNA^Ser^ in two different modes (6,7,9). Interestingly, *Sc*Trm140 is expressed in fusion with an upstream actin-binding motif by a programmed +1 frameshift. However, in the fission yeast *Schizosaccharomyces pombe*, two separate genes encode two m^3^C32 methyltransferases, Trm140 (*Sp*Trm140) and Trm141 (*Sp*Trm141), without the actin-binding motif (10). Accordingly, *Sp*Trm140 is no longer a dual-specificity enzyme but modifies only tRNA^Thr^, while tRNA^Ser^ is complementarily modified by *Sp*Trm141 (10). In mammalian cells, mouse Mettl2 and human METTL2A and METTL2B are homologous to *Sc*Trm140/*Sp*Trm140 and have been shown to be responsible for m^3^C32 formation in tRNA^Thr^ species (8). Recently, it has been shown that an additional cofactor, DALRD3, must interact with human METTL2A and/or METTL2B to induce m^3^C32 formation in human tRNA^Arg^(CCU) and tRNA^Arg^(UCU) species (11), although the interaction pattern and the precise role of each component in modification are still unknown. In addition, *Sp*Trm141-homologous mouse Mettl6 catalyzes m^3^C32 formation in tRNA^Ser^ species. Interestingly, both *Sp*Trm141 and Mettl6 interact with seryl-tRNA synthetase (SerRS) (8,9), suggesting that interaction with SerRS is an evolutionarily inherent ability of *Sp*Trm141/Mettl6. The m^3^C32 modification activity of *Sp*Trm141 is greatly stimulated by the presence of SerRS *in vitro* (9). However, whether *Sp*Trm141 or Mettl6 alone could mediate m^3^C32 biogenesis is not yet known, and the precise interaction mode and role of either *Sp*Trm141/Mettl6 or SerRS in tRNA binding are not fully understood. In addition, the biological function of m^3^C32 is poorly understood. Considering its localization in the anticodon loop, it possibly influences precise pairing between codon and anti-codon and/or biogenesis of ANG-mediated tRNA-derived fragments, as revealed by inhibition effect of m^5^C formation at C38 (12).

Although genetic data have clearly revealed the above m^3^C32 methyltransferases, the reconstitution of m^3^C32 activity using tRNA transcripts *in vitro* has not been successfully realized (7). Instead, *in vitro* m^3^C32 activity was achieved using tRNAs purified from a *Sc*Trm140 gene deletion strain, suggesting that other modifications prior to m^3^C32 are prerequisites (7). Indeed, genetic studies have clearly demonstrated that t^6^A at position 37 (t^6^A37, catalyzed by Sua5 and KEOPS in yeasts) (13,14) or i^6^A at position 37 (i^6^A37, catalyzed by MOD5 in yeasts) (15) in specific tRNA substrates significantly triggers m^3^C32 biogenesis (9,10), giving an exciting example of a tRNA modification circuit (16). Therefore, to determine the tRNA recognition pattern of *Sc*Trm140, different tRNA mutants were expressed *in vivo*, and the modification status at position 32 was monitored by primer extension assays, which confirmed the importance of t^6^A37 or i^6^A37 modification and revealed the key nucleotides in tRNAs (9). However, this assay does not directly determine methylation by *Sc*Trm140, limiting the full understanding of the contributions of other nucleotides of tRNA and of the key amino acids of the methyltransferase or cofactor in m^3^C biogenesis.

On the other hand, the methylation of nucleotides in mRNA, such as 6-methyladenosine (m^6^A) and 5-methylcytidine (m^5^C), plays important roles in gene expression at multiple levels by influencing RNA structure and interactions within the ribosome or by recruiting specific binding proteins that communicate with other signaling pathways in physiological or pathological processes (17–19). Indeed, in addition to its presence in tRNA, m^3^C is present in mRNA and is suggested to be catalyzed by *Sc*Trm140/*Sp*Trm141/Mettl2a/Mettl6-homologous Mettl8 (8), despite a recent work reporting a dramatically lower abundance of m^3^C in mRNA than in tRNA (20). However, neither the specific recognition of mRNA and the mechanism of catalysis by Mettl8 nor the potential physiological or pathological role of m^3^C in mRNA has yet been clearly established. Therefore, studies of the substrate selection mechanism by Mettl8-homologous tRNA m^3^C32 methyltransferases should help to understand the mRNA m^3^C modification mechanism.

To understand why mammalian cells need more than one m^3^C32 methyltransferase and how various homologous enzymes discriminate specific tRNA substrates, in this work, using human tRNA m^3^C32 methyltransferases (METTL2A, METTL2B and METTL6) as models, we studied their gene expression and cellular localization; we further purified human METTL2A, METTL2B and METTL6 and prepared tRNA transcripts with and without t^6^A37 modification. We successfully reconstituted the robust m^3^C32 activities of both METTL2A and METTL6 *in vitro*, showed that METTL2B exhibited only limited methyltransferase activity *in vitro* and further provided a detailed tRNA selection mechanism by both enzymes.

## MATERIALS AND METHODS

### Materials

Anti-FLAG (F7425), anti-Myc (M4439) and anti-GAPDH (G8795) antibodies were purchased from Sigma (St. Louis, MO, USA). Anti-His_6_ (AE003) was purchased from Abclonal (Shanghai, China). [^3^H] SAM, [^3^H] Arg, [^14^C] Thr and [^14^C] Ser were obtained from Perkin Elmer Inc. (Waltham, MA, USA). Dynabeads Protein G, MitoTracker and Lipofectamine 2000 transfection reagent were obtained from Thermo Scientific (Waltham, MA, USA). Primers were synthesized in Biosune (Shanghai, China), and DNA sequencing was performed by Tsingke (Shanghai, China).

### Plasmid construction, expression and protein purification

Genes encoding METTL2A (UniProt No. Q96IZ6), METTL2B (UniProt No. Q6P1Q9), METTL6 (UniProt No. Q8TCB7) and SerRS (UniProt No. P49591) were amplified from cDNA obtained by reverse transcription of total RNA from human HEK293T cells. For gene expression in HEK293T cells, *METTL2A* and *METTL2B* were inserted between the Hind III and Xho I sites of pCMV-3Tag-3A and pCMV-3Tag-4A, and *METTL6* was inserted between the Hind III and Xho I sites of pcDNA3.1. For gene expression in *E. coli*, *METTL2A* and *METTL2B* were inserted between the Sac I and Not I sites of pRSFDuet1 with an N-terminal His_6_ tag, respectively. *METTL6* was inserted between the SacI and NotI sites of pRSFDuet1 with an N-terminal His_6_ tag. *SerRS* was inserted between the Nde I and Xho I sites of pET22b with a C-terminal His_6_ tag. The primers used for cloning are listed in Supplementary Table S2. The METTL2A, METTL2B, METTL6 and SerRS genes were expressed in *Escherichia coli* BL21 (DE3) cells and induced with 200 μM isopropyl β-d-1-thiogalactopyranoside (IPTG) when the initial cell culture reached an absorbance at 600 nm (*A*_600_) of 0.6, and transformants were cultured overnight at 18°C. Protein purification from *Escherichia. coli* transformants was performed with a procedure described in a previous report (21). Protein concentration was determined using a Protein Quantification Kit (BCA Assay, Beyotime, Shanghai, China) according to the manufacturer's instructions.

### tRNA gene cloning and transcription

Genes encoding human cytoplasmic (hc) tRNA^Thr^(AGU, CGU, UGU), tRNA^Ser^(GCU), tRNA^Arg^(CCU, UCU), tRNA^Asn^(GUU), tRNA^Met^(e) and *E. coli* tRNA^Thr^(UGU) (*Ec*tRNA^Thr^) were incorporated into the pTrc99b plasmid. tRNA transcripts were obtained by *in vitro* T7 RNA polymerase transcription as described previously (22,23). The overexpression and purification of *Ec*tRNA^Thr^ from *E. coli* have been described in previous reports (24,25). tRNA gene mutagenesis was performed according to the protocol provided with the KOD-plus mutagenesis kit. The primers used for template preparation are listed in Supplementary Table S2.

### Determination of amino acid accepting activities

The amino acid accepting activities of various tRNAs were determined in the following reactions. A 40 μl reaction mixture containing 50 mM Tris–HCl, pH 7.5, 20 mM KCl, 10 mM MgCl_2_, 2 mM DTT, 4 mM ATP, 20 μM [^14^C] Thr and 2.5 μM hctRNA^Thr^ was incubated with 2 μM mThrRS; a 40 μl reaction mixture containing 50 mM Tris-HCl, pH 7.5, 20 mM KCl, 10 mM MgCl_2_, 2 mM DTT, 4 mM ATP, 20 μM [^14^C] Ser and 2.5 μM hctRNA^Ser^(GCU) was incubated with 2 μM SerRS; a 40 μl reaction mixture containing 50 mM Tris–HCl, pH 7.5, 80 mM KCl, 12 mM MgCl_2_, 2 mM DTT, 4 mM ATP, 20 μM [^3^H] Arg and 2.5 μM hctRNA^Arg^ was incubated with 2 μM ArgRS. At time intervals ranging between 15 and 60 min, aliquots of the reaction solution were added to Whatman filter pads and subsequently processed in a similar procedure with aminoacylation assays (26).

### t^6^A activity assay and t^6^A modification of tRNAs

The t^6^A modification reaction was performed at 37°C in a 40 μl reaction mixture containing 50 mM Tris–HCl (pH 8.0), 200 mM NaCl, 15 mM MgCl_2_, 5 mM MnCl_2_, 50 mM NaHCO_3_, 5 mM DTT, 4 mM ATP, 100 μM [^14^C] Thr, 10 μM hctRNAs or variants and 2 μM Sua5 and KEOPS.

Modification of tRNAs or variants with t^6^A was performed as follows: a 200 μl reaction mixture containing 50 mM Tris–HCl (pH 8.0), 200 mM NaCl, 15 mM MgCl_2_, 5 mM MnCl_2_, 50 mM NaHCO_3_, 5 mM DTT, 4 mM ATP, 1 mM Thr, 50–100 μg of tRNAs or variants and 5 μM Sua5 and KEOPS. The reaction was incubated at 37°C for 1 h, and the t^6^A-modified tRNA was purified by phenol and chloroform and precipitated by EtOH with NaAc overnight at –20°C. The t^6^A-modified tRNA concentration was determined by denaturing UREA-PAGE based on linear curves from tRNA transcript samples with known concentrations.

### tRNA methylation assay

The methylation reactions were performed at 37°C in a reaction mixture containing 50 mM Tris–HCl (pH 7.5), 20 mM KCl, 10 mM MgCl_2_, 10 mM spermidine, 10 mM DTT, 20 μM [^3^H] SAM, 5 μM transcribed, t^6^A-modified or overexpressed tRNAs, and 1 μM METTL2A, METTL2B or METTL6. At time intervals ranging between 5 and 15 min, aliquots were removed to Whatman filter pads and processed as described above.

### LC-MS/MS analysis of t^6^A and m^3^C modified tRNA

One microgram of hctRNA^Thr^(AGU), t^6^A-hctRNA^Thr^(AGU), or m^3^C-t^6^A-hctRNA^Thr^(AGU) was completely hydrolyzed by benzonase, phosphodiesterase I, and bacterial alkaline phosphatase in a 60 μl reaction containing 20 mM NH_4_Ac (pH 5.2) at 37°C for 24 h. One microliter of the solution was then applied to LC–MS/MS analysis. The nucleosides were separated by HPLC on a C18 column (Agilent Zorbax Eclipse Plus C18, 2.1 50 mm, 1.8 mm) and then detected by a triple-quadrupole mass spectrometer (Agilent 6495 QQQ) in positive ion multiple reaction-monitoring mode. Mass transitions from *m*/*z* 413.1 to 281.1 (t^6^A) and *m*/*z* 258.1 to 126.1 (m^3^C) were monitored and recorded.

### Cell culture, transfection and co-immunoprecipitation (Co-IP)

HEK293T cells were cultured in Dulbecco's modified Eagle's medium supplemented with 10% fetal bovine serum in a 37°C incubator with 5% CO_2_ at a confluence of 70% before transfection using Lipofectamine 2000 transfection reagent according to the manufacturer's protocol. At 24 h after transfection, cells were harvested, washed with ice-cold phosphate-buffered saline (PBS) three times, and lysed with 1 ml of ice-cold lysis buffer (50 mM Tris–HCl, pH 7.5, 150 mM NaCl, 5 mM ethylenediaminetetraacetic acid, 1% Triton X-100) supplemented with a protease inhibitor cocktail for 15 min at 4°C with rotation. Co-IP was performed as described previously (27,28).

### Western blotting

Protein samples were separated on a 10% separating gel by SDS-PAGE and transferred to a methanol-activated polyvinylidene fluoride (PVDF) membrane, which was then blocked with 5% milk in PBST for 1 h at room temperature. Immunoblotting was performed using anti-FLAG, anti-Myc or anti-HA antibodies overnight, followed by incubation with secondary antibodies, and detected as described previously (27,28)

### Immunofluorescence

HEK293T cells were transfected with specific plasmids. After 24 h, cells were stained with MitoTracker for 30 min and then fixed in 4% paraformaldehyde containing PBS for 30 min at room temperature. Fixed cells were blocked in PBS plus 0.1% Triton X-100 buffer containing 5% BSA and incubated with the primary antibody overnight at 4°C. The cells were immunostained with Alexa Fluor 488-conjugated secondary antibody in PBS for 2 h and the nuclear counterstain DAPI for 5 min at room temperature. Fluorescence images were captured with a Leica TCS SP8 STED confocal microscope.

## RESULTS

### Both METTL2A and METTL2B are expressed *in vivo* and located in the cytoplasm

In mammalian cells, METTL2A, METTL2B and METTL6 have been shown to participate in tRNA^Thr^ and tRNA^Ser^ m^3^C32 modification (8). Primary sequence analysis showed that the three human enzymes, together with *Sc*Trm140 and *Sp*Trm141, share a conserved C-terminal S-adenosyl methionine (SAM) binding domain (CTD), while a striking difference exists in the N-terminal domain (NTD) with unassigned function (Supplementary Figure S2). Compared with *Sc*Trm140, human METTL2A and METTL2B have a large insertion (approximately 68 aa in length) in the NTD, while *Sc*Trm141 and human METTL6 display a truncated NTD in the N-terminus. However, all the enzymes retain a conserved ‘FFKDR’ motif with an unknown role in the NTD (Supplementary Figure S2; Supplementary Figure S3A).

In humans, *METTL2A* (gene ID 339175) and *METTL2B* (gene ID 55798) are encoded by two separate genes located on chromosomes 17 and 7, respectively. However, only one gene, *Mettl2* (gene ID 52686) on chromosome 11, encodes a single Mettl2 for m^3^C32 modification in mice. Due to the nearly identical genomic and protein sequences of human METTL2A and METTL2B (see text below), the detailed evolutionary path between the two human genes is unclear. Considering protein sequences (both 378 residues in length), only six different sites are present among the two enzymes. Three positions harbor amino acids with similar side chain properties (Val^12^, Ile^266^ and Met^288^ in METTL2A *vs*. Ile^12^, Val^266^ and Val^288^ in METTL2B), while the other three positions have completely different residues (Arg^26^, Pro^124^ and Leu^155^ in METTL2A *vs*. Ser^26^, Cys^124^ and Pro^155^ in METTL2B) (Supplementary Figure S3A).

We initially explored whether one or two genes are expressed in human cells by using liquid chromatography-tandem mass spectrometry (LC-MS) analysis of the whole cell lysis (WCL) of HEK293T cells to capture METTL2A- or METTL2B-specific peptides. Indeed, peptides spanning the same region in both METTL2A (AGSYPEGAPAVLADKR) and METTL2B (AGSYPEGAPAILDKR) were clearly detected (Supplementary Figure S3B). These data definitely showed that both METTL2A and METTL2B genes are expressed *in vivo*.

Furthermore, we introduced a gene encoding a C-terminal FLAG-tagged METTL2A (METTL2A-FLAG) or METTL2B (METTL2B-FLAG) separately into HEK293T cells. Immunofluorescence (IF) assays showed that METTL2A-FLAG and METTL2B-FLAG were both distributed in the cytoplasm (Supplementary Figure S3C). No clear fluorescence signal was observed in the mitochondria (Supplementary Figure S3C).

Subsequently, we purified METTL2A from *E. coli* cells to high homogeneity (Supplementary Figure S4A). The calculated molecular mass of purified METTL2A together with the His_6_-tag should be 45.4 kDa. Its molecular mass was determined to be 34.3 kDa by gel filtration analysis with Superdex S200 based on the elution volumes of three standard proteins, apoferritin (443 kDa), yeast alcohol dehydrogenase (150 kDa) and bovine serum albumin (BSA, 66 kDa) (Supplementary Figure S4B, C). Considering that the elution volume of METTL2A was even larger than that of BSA with the smallest molecular mass among the three standards, to more accurately determine its molecular mass, a similar analysis was also performed using Superdex S75, and the molecular mass was determined to be 48.5 kDa (Supplementary Figure S4D, E) based on four standard proteins, conalbumin (75 kDa), ovalbumin (44 kDa), ribonuclease A (RNase A, 13.7 kDa) and aprotinin (6.5 kDa). These results suggested that purified METTL2A was a monomer in solution.

The above data collectively revealed that both the METTL2A and METTL2B genes are readily expressed and that METTL2A and METTL2B are located in the cytoplasm.

### t^6^A37 is essential for m^3^C32 biogenesis of tRNA^Thr^ by METTL2A

**Figure 1.**
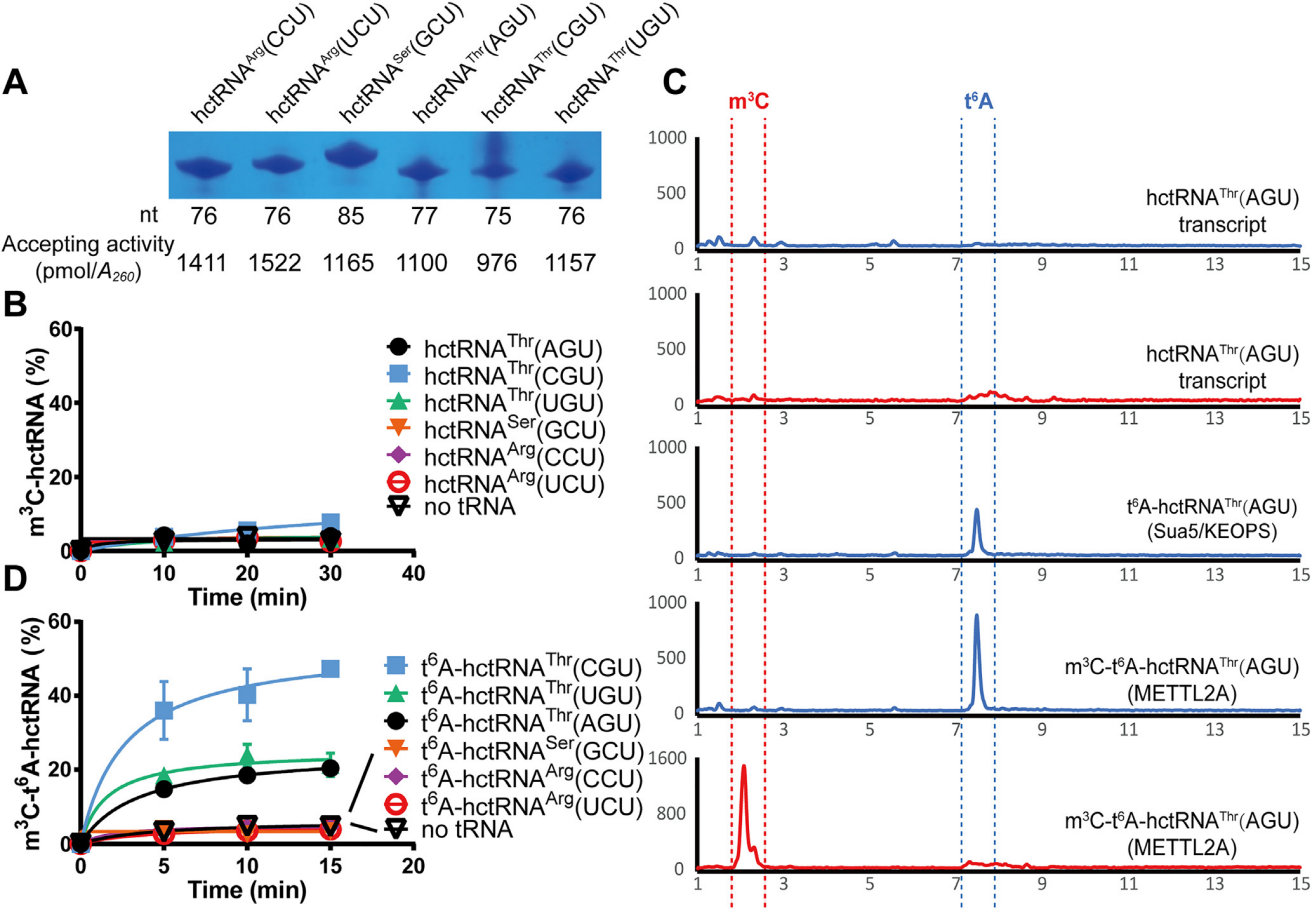
t^6^A37 is essential for the m^3^C32 modification of hctRNA^Thr^. (**A**) Urea gel separation of six tRNA transcripts. Their lengths and amino acid accepting activities are indicated below. (**B**) Time-course curves of the m^3^C modification of six hctRNA transcripts by METTL2A. (**C**) LC–MS/MS analysis of the digested products of the hctRNA^Thr^(AGU) transcript, modification products by Sua5/KEOPS and subsequently by METTL2A. (**D**) Time-course curves of the m^3^C modification of the six t^6^A37-modified hctRNAs by METTL2A. Data represent averages of two independent experiments (A) or three independent experiments (B) and the corresponding standard deviation.

m^3^C32 is present in tRNA^Thr^, tRNA^Ser^ and tRNA^Arg^ in human cells (5). These tRNAs, including hctRNA^Thr^(AGU, CGU, UGU), hctRNA^Ser^(GCU) and hctRNA^Arg^(CCU, UCU), were prepared by *in vitro* T7 run-off transcription (Figure 1A). To validate correct tRNA folding and quality, their corresponding aminoacyl-tRNA synthetases (aaRSs), including mouse cytoplasmic threonyl-tRNA synthetase (ThrRS) (26), human cytoplasmic SerRS and arginyl-tRNA synthetase (ArgRS) (29), were purified. The amino acid accepting activities of these tRNAs were approximately 1000–1500 pmol/*A*_260_ (Figure 1A), indicating that the tRNAs were correctly folded and of high quality.

Subsequently, we used METTL2A and these tRNAs to reconstitute m^3^C32 modification activity *in vitro*. None of these tRNAs were modified by METTL2A (Figure 1B) [modification of hctRNA^Thr^(CGU) was negligible, if any], indicating that purified METTL2A is inactive *in vitro*, or is active but tRNA transcripts are not suitable substrates or needs a cofactor for catalysis. *Sc*Trm140 recognizes tRNA^Thr^ substrates via a sequence element including t^6^A37 (9). To understand whether t^6^A37 is a prerequisite for m^3^C32 formation in humans, the *S. cerevisiae* t^6^A modification machinery, including Sua5 and KEOPS, was purified (22). Sua5/KEOPS was able to efficiently modify all six human cytoplasmic tRNA transcripts *in vitro* (see text below). The tRNA^Thr^(CGU) modified by Sua5/KEOPS was collected, digested with benzonase and analyzed by liquid chromatography coupled with electrospray ionization tandem mass spectrometry (LC-MS/MS). t^6^A37 was readily detected (Figure 1C), suggesting efficient modification of t^6^A37 by Sua5/KEOPS. Then, methylation assays clearly showed that METTL2A was able to introduce m^3^C32 only into the three t^6^A-hctRNA^Thr^ species but not into t^6^A-hctRNA^Arg^(CCU), -hctRNA^Arg^(UCU) or -hctRNA^Ser^(GCU) (Figure 1D). However, the modification levels of the three tRNA^Thr^ species were different, with tRNA^Thr^(CGU) having the highest efficiency. In addition, LC-MS/MS analysis confirmed that the m^3^C32 moiety was readily decorated in the modified hctRNA^Thr^(CGU) products (Figure 1C).

Thus, we successfully reconstituted m^3^C32 modification activity by METTL2A and revealed that METTL2A alone could modify only tRNA^Thr^ but not tRNA^Arg^ and tRNA^Ser^, which requires t^6^A37 as a prerequisite.

### G35 is a determinant of METTL2A for m^3^C32 activity reconstitution

**Figure 2.**
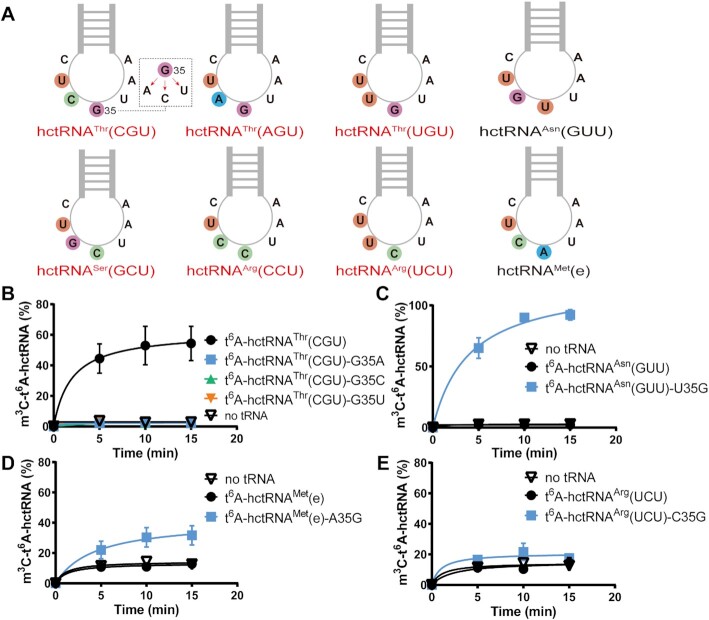
G35 is a determinant of m^3^C biogenesis. (**A**) A schema showing the sequences of anticodon loops of six hctRNAs with t^6^A and m^3^C32 modifications (indicated in red) and two hctRNAs [hctRNA^Asn^(GUU) and hctRNA^Met^(e)] with only t^6^A modifications. The m^3^C modification levels of t^6^A-hctRNA^Thr^(CGU) (black filled circles) and t^6^A-hctRNA^Thr^(CGU)-G35A (blue filled squares), -G35C (green filled triangles), and -G35U (orange filled inverted triangles) (**B**); of t^6^A-hctRNA^Asn^(GUU) (green filled circles) and t^6^A-hctRNA^Asn^(GUU)-U35G mutant (blue filled squares) (**C**); of t^6^A-hctRNA^Met^(e) (black filled circles) and t^6^A-hctRNA^Met^(e)-U35G (blue filled squares) (**D**); and of t^6^A-hctRNA^Arg^(UCU) (black filled circles) and t^6^A-hctRNA^Arg^(UCU)-U35G (blue filled squares) (**E**) by METTL2A. Data represent averages of three independent experiments and the corresponding standard deviation.

We further explored how METTL2A discriminates among different tRNA substrates. Due to the localization of position 32 in the anticodon loop, the various anticodon loops of three hctRNA^Thr^s, two hctRNA^Arg^s and hctRNA^Ser^ were checked. Each of the hctRNA^Thr^s, with C34, A34 or U34, could be modified, suggesting that position 34 is not a key site for modification. Among other bases in the loop, only position 35 is divergent among these tRNAs, with G35 in all tRNA^Thr^s (Figure 2A). To understand its potential importance, G35 of hctRNA^Thr^(CGU) (with the highest m^3^C32 modification efficiency) was then mutated to A35, C35 or U35. The tRNA mutants were t^6^A-modified by Sua5/KEOPS to comparable levels, suggesting that G35 is not an identity element in t^6^A modification (Supplementary Figure S5A), which was consistent with observations with the human mitochondrial t^6^A modification enzyme OSGEPL1 (22). After the preparation of t^6^A-modified tRNA^Thr^(CGU) mutants, methylation determination clearly revealed that m^3^C32 was no longer formed in the mutants (Figure 2B), suggesting that G35 was a determinant in m^3^C32 biogenesis by METTL2A.

Subsequently, in addition to hctRNA^Arg^(UCU) (with C35), we also transcribed hctRNA^Asn^(GUU) (with U35) and hctRNA^Met^(e) (with A35), which are used to decode codons starting with A (ANN codons) and are supposed to be modified with t^6^A (note that hctRNA^Asn^(GUU) and hctRNA^Met^(e) do not contain m^3^C32 in human cells) (30). The nucleotides at position 35 of these tRNAs were also changed to G35. No impairment (instead an increase in hctRNA^Asn^(GUU) and hctRNA^Met^(e)) was observed in t^6^A modification by Sua5/KEOPS with wild-type and mutant tRNAs (Supplementary Figure S5B–D). However, different effects of the presence of G35 in various tRNA species were monitored; both t^6^A-modified hctRNA^Asn^(GUU)-U35G and hctRNA^Met^(e)-A35G clearly gained an m^3^C32 modification (Figure 2C, D), while m^3^C32 was only negligibly (if at all) introduced into hctRNA^Arg^(UCU)-C35G (Figure 2E), implying that other elements in addition to t^6^A37 and G35 also critically control m^3^C32 formation by METTL2A.

Therefore, the above evidence showed that G35 is a critical element in m^3^C32 formation in tRNA^Thr^ species; introducing only a single G35 into a non-m^3^C tRNA [hctRNA^Asn^(GUU) or hctRNA^Met^(e)] could confer the capacity to be modified by METTL2A.

### G35 and t^6^A37 are insufficient for m^3^C modification

**Figure 3.**
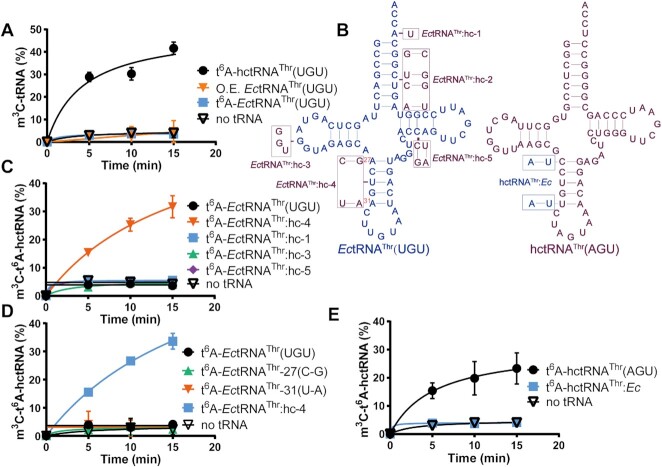
Anticodon stems harbor critical elements for m^3^C32 modification by METTL2A. (**A**) m^3^C modification levels of t^6^A-hctRNA^Thr^(UGU) (black filled circles), t^6^A-*Ec*tRNA^Thr^(UGU) (blue filled squares) and overexpressed (O.E.) *Ec*tRNA^Thr^(UGU) (orange filled inverted triangles) by METTL2A. (**B**) Secondary structures of *Ec*tRNA^Thr^(UGU) (left) and hctRNA^Thr^(AGU) (right) showing the construction of five *Ec*tRNA^Thr^ mutants and one hctRNA^Thr^(AGU) mutant. m^3^C modification levels of t^6^A-*Ec*tRNA^Thr^(UGU) (black filled circles) and t^6^A-*Ec*tRNA^Thr^:hc-1 (blue filled squares), :hc-3 (green filled triangles), :hc-4 (orange filled inverted triangles) and :hc-5 (purple filled diamond) (**C**); of t^6^A-*Ec*tRNA^Thr^(UGU) (black filled circles) and t^6^A-*Ec*tRNA^Thr^:hc-4(blue filled squares), -27(C-G) (green filled triangles) and -31(U-A) (orange filled inverted triangles) (**D**); and of t^6^A-hctRNA^Thr^(AGU) (black filled circles) and t^6^A-hctRNA^Thr^(AGU):*Ec* (blue filled squares) (**E**) by METTL2A. *Ec*tRNA^Thr^:hc-2 could not be modified with t^6^A37 and was thus not included in the methylation assay. Data represent averages of three independent experiments (A, C) or two independent experiments (D, E) and the corresponding standard deviation.

The above data from hctRNA^Arg^(UCU)-C35G showed that t^6^A37 and G35 alone are insufficient to confer m^3^C32 modification in specific tRNAs. t^6^A37 and G35 are present in *E. coli* tRNA^Thr^ species; however, m^3^C32 is absent in bacterial tRNAs due to the lack of m^3^C32 methyltransferase. To study whether METTL2A has the ability to introduce m^3^C32 to bacterial tRNA species, we prepared a t^6^A-modified *E. coli* tRNA^Thr^(UGU) (*Ec*tRNA^Thr^) transcript using Sua5/KEOPS. However, we found that t^6^A-containing *Ec*tRNA^Thr^ was not modified by METTL2A (Figure 3A). To understand whether other modifications of *Ec*tRNA^Thr^ were required for efficient reconstitution, we overexpressed and purified *Ec*tRNA^Thr^ from the *E. coli* MT102 strain. However, native *Ec*tRNA^Thr^ was likewise not a substrate of METTL2A. Notably, the t^6^A-containing hctRNA^Thr^(UGU) transcript was clearly modified by METTL2A (Figure 3A). These results suggested that the tRNA sequence is the primary element leading to *Ec*tRNA^Thr^ hypomodification.

Based on the sequence of *Ec*tRNA^Thr^ and hctRNA^Thr^(AGU) (the two tRNAs were compared because they display the highest sequence identity), we designed five *Ec*tRNA^Thr^ mutants by replacing some elements with their counterparts in hctRNA^Thr^(AGU), including *Ec*tRNA^Thr^:hc-1 (with A73U), :hc-2 (amino acid acceptor stem swapping), :hc-3 (^17^AGG^19^ replaced by ^17^GGU^19^ in the D-loop), :hc-4 (anticodon stem swapping) [note that the two stems differ only in two base pairs, A27-U43/A31-U39 in *Ec*tRNA^Thr^ versus C27-G43/U31-A39 in hctRNA^Thr^(AGU)], and :hc-5 (C51-G63/C62 replaced by G51-C63/U62 in the TψC-stem) (Figure 3B). However, *Ec*tRNA^Thr^:hc-2 was defective in t^6^A37 modification by Sua5/KEOPS, while the other mutants were modified with t^6^A37 to comparable levels (Supplementary Figure S5E). The determination of m^3^C32 activity showed that METTL2A indeed gained the ability to modify only *Ec*tRNA^Thr^:hc-4 (Figure 3C), highlighting the determinant role of the anticodon stem. To further verify which base pairs play a key role in *Ec*tRNA^Thr^:hc-4, we obtained two *Ec*tRNA^Thr^ mutants based on *Ec*tRNA^Thr^:hc-4 [*Ec*tRNA^Thr^-27(C–G) and *Ec*tRNA^Thr^-31(U–A)]. m^3^C32 activity determination showed that both the C27–G43 and U31–A39 base pairs are needed to confer modification on *Ec*tRNA^Thr^ (Supplementary Figure S5F, Figure 3D). Conversely, we replaced the anticodon stem of hctRNA^Thr^(AGU) with that of *Ec*tRNA^Thr^ (Figure 3B), and the resultant hctRNA^Thr^(AGU):*Ec* displayed impaired but obvious t^6^A37 modification by Sua5/KEOPS (Supplementary Figure S5G) but was hypomodified by METTL2A (Figure 3E).

The above results clearly showed that in addition to G35 and t^6^A37, sequences in the anticodon stem are critical elements in determining the m^3^C32 activity of METTL2A. However, we found that the anticodon stem sequences are not completely conserved among human tRNA^Thr^ species, suggesting that they likely work collaboratively with anticodon loops and/or other elements to determine m^3^C32 levels in specific tRNAs, which is likely why the three human tRNA^Thr^ species displayed different m^3^C32 modification levels *in vitro* (Figure 1D).

### The rationale of hctRNA^Ser^(GCU) not modified by METTL2A

**Figure 4.**
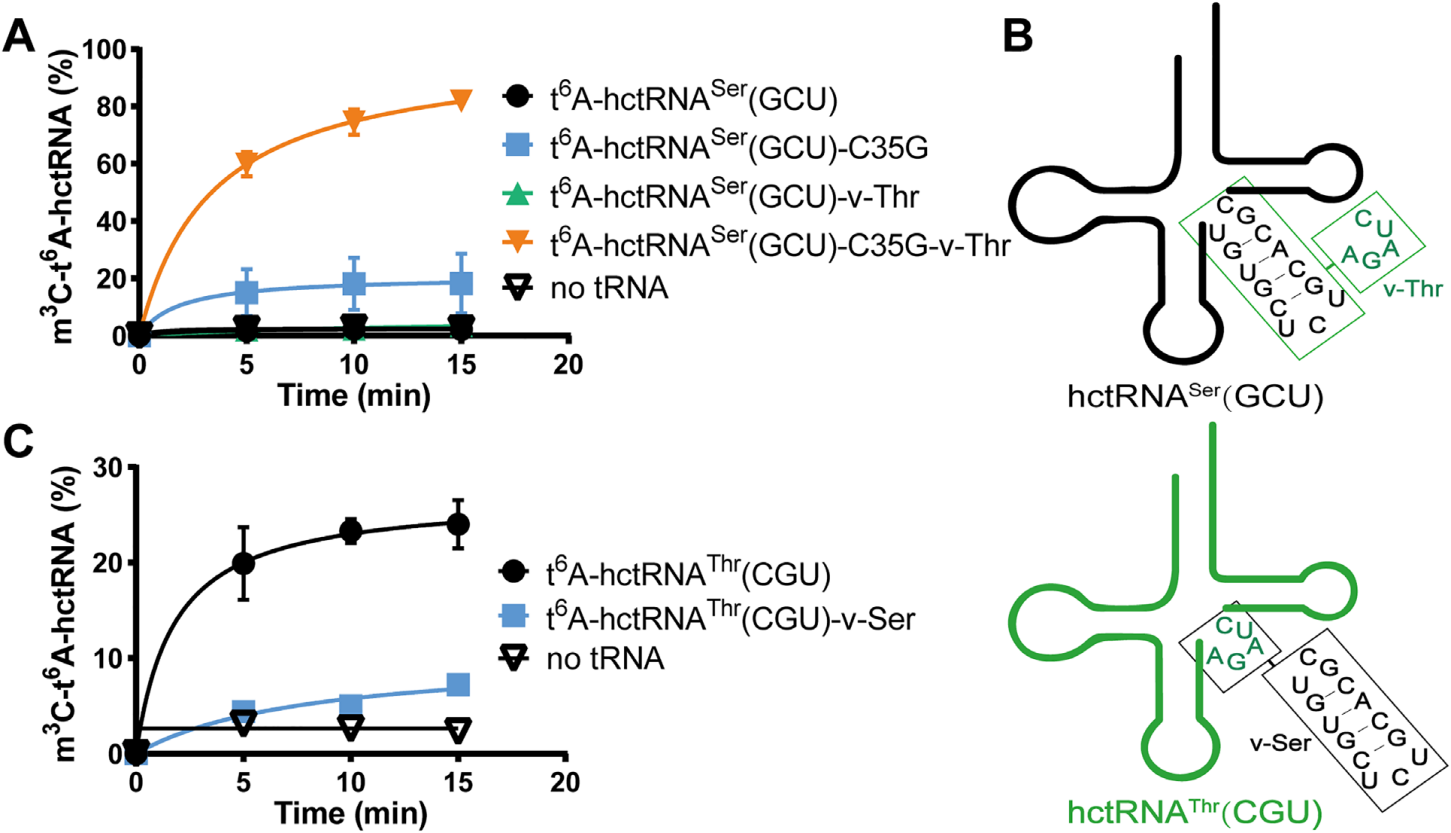
C35 and the long variable arm prevent hctRNA^Ser^(GCU) from being modified by METTL2A. m^3^C32 modification levels of t^6^A-tRNA^Ser^(GCU) (black filled circles), t^6^A-tRNA^Ser^(GCU)-C35G (blue filled squares), -v-Thr (orange filled triangles) and -C35G-v-Thr (green filled inverted triangles) (**A**) and of t^6^A-hctRNA^Thr^(CGU) (black filled circles) and t^6^A-hctRNA^Thr^(CGU)-v-Ser (blue filled squares) by METTL2A (**C**). (**B**) Schematic diagram showing the construction of various hctRNA^Ser^(GCU) and hctRNA^Thr^(CGU) variable arm mutants. Data represent averages of three independent experiments (A) or two independent experiments (**C**) and the corresponding standard deviation.

The above data clarified the key role of G35 and t^6^A37 in determining the m^3^C32 modification status by METTL2A. We have shown that t^6^A-hctRNA^Ser^(GCU) was not modified by METTL2A *in vitro*. Therefore, we changed C35 of hctRNA^Ser^(GCU) to G35, and the mutant hctRNA^Ser^(GCU)-C35G exhibited a similar level of t^6^A modification to wild-type tRNA (Supplementary Figure S5H). Strikingly, the data showed that METTL2A could indeed introduce m^3^C32 into hctRNA^Ser^(GCU)-C35G (Figure 4A), confirming the critical role of G35 in determining the m^3^C32 modification specificity of METTL2A.

hctRNA^Ser^(GCU) differs from hctRNA^Thr^ predominantly in the long variable arm. To understand whether the long variable arm was also a negative element in m^3^C32 activity by METTL2A, we replaced hctRNA^Ser^(GCU) (^44^UGUGCUCUGCACGC^48^) with hctRNA^Thr^(CGU) (^44^AGAUC^48^) (Figure 4B). Despite the lack of impairment in t^6^A modification (Supplementary Figure S5H), however, the resultant hctRNA^Ser^(GCU)-v-Thr was still hypomodified by METTL2A (Figure 4A). In sharp contrast, in the C35G context, hctRNA^Ser^(GCU)-C35G-v-Thr showed a clearly and robustly greater m^3^C32 modification level than hctRNA^Ser^(GCU)-C35G (Figure 4A). In parallel, we changed the variable loop of hctRNA^Thr^(CGU) to that of hctRNA^Ser^(GCU) (Figure 4B). No impairment in t^6^A modification level was observed (Supplementary Figure S5I); however, m^3^C32 modification of the mutant hctRNA^Thr^(CGU)-v-Ser was significantly decreased to only slightly higher than the basal level (Figure 4C).

Taken together, these data elucidated that the absence of G35 and the presence of the long variable arm of hctRNA^Ser^(GCU) precluded its modification by METTL2A.

### METTL2B exhibited little m^3^C32 modification activity *in vitro*

Both the METTL2A and METTL2B genes are well expressed *in vivo* and have the same cytoplasmic distribution, eliciting the question of whether they display similar and redundant m^3^C32 modification activity. Thus, we purified METTL2B to high homogeneity (Supplementary Figure S6A). Unexpectedly, methyltransferase activity determination showed that the activity of METTL2B was only approximately 1/10 of that of METTL2A (Supplementary Figure S6B). METTL2A contains three sites (Arg^26^, Pro^124^ and Leu^155^) that exhibit totally different side chain properties from their counterparts in METTL2B (Ser^26^, Cys^124^ and Pro^155^). Thus, three single-point mutants, METTL2A-R26S, -P124C and -L155P, were constructed and purified (Supplementary Figure S6A). Subsequent methylation measurement showed that the activities of both METTL2A-R26S and -L155P were reduced to approximately half that of the wild-type enzyme, and the activity of METTL2A-P124C was as low as that of METTL2B (Supplementary Figure S6B). These results suggested that several natural amino acids in METTL2B, especially Cys^124^, likely determined its low m^3^C32 methylation activity in comparison with METTL2A.

### METTL6 is located in the cytoplasm and nucleus and interacts with SerRS

A previous report has shown that mouse tRNA^Ser^ species are modified by Mettl6 (8). We overexpressed a gene encoding C-terminal FLAG-tagged human METTL6 (METTL6-FLAG) in HEK293T cells, and IF analysis showed that METTL6-FLAG was distributed in both the cytoplasm and nucleus. Its possible mitochondrial localization was not observed (Supplementary Figure S7A). Furthermore, previous studies have shown that yeast SerRS stimulates the activity of *Sc*Trm140 (9). METTL6-FLAG and a C-terminal Myc-tagged SerRS (SerRS-Myc) were coexpressed in HEK293T cells. By using anti-FLAG antibodies to perform Co-IP, SerRS-Myc could be precipitated with METTL6-FLAG (Supplementary Figure S7B). To understand whether the interaction is direct or indirect by relying on the presence of RNA or DNA, we then digested the DNA or RNA of whole cell lysates by DNase I or RNase A prior to immunoprecipitation. The results suggested that the interaction between METTL6 and SerRS was disrupted by RNase treatment; however, DNase I treatment was unable to abolish the interaction (Supplementary Figure S7B), suggesting that the interaction of METTL6 with SerRS depends on RNA (likely tRNA substrates).

### SerRS is essential for the m^3^C32 biogenesis of hctRNA^Ser^(GCU) by METTL6

**Figure 5.**
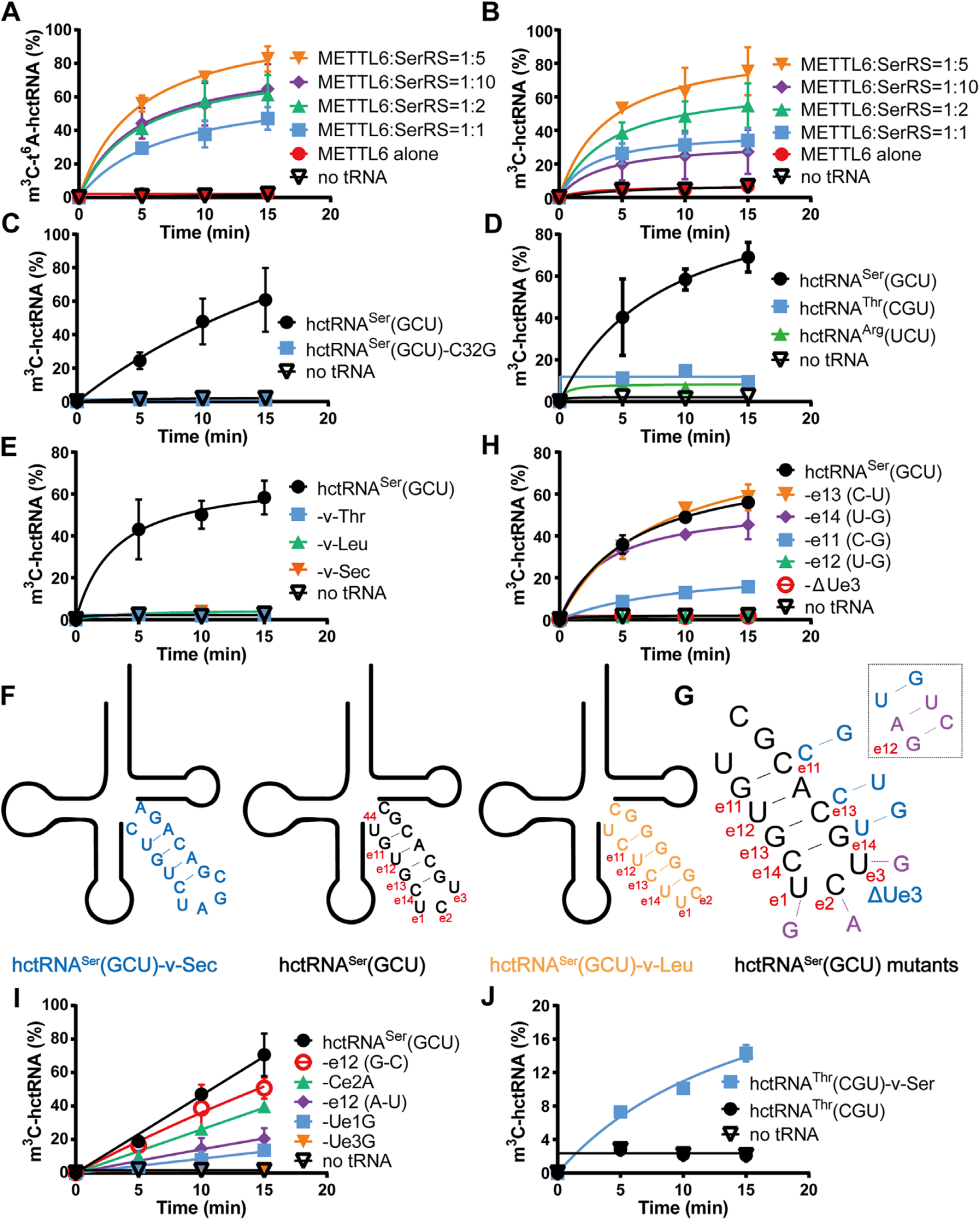
m^3^C modification activity of METTL6 for hctRNA^Ser^(GCU) requires the presence of SerRS and the long variable arm. m^3^C modification levels of t^6^A-hctRNA^Ser^(GCU) (**A**) and hctRNA^Ser^(GCU) transcripts (**B**) by METTL2A without SerRS (red filled circles) and with increasing amounts of SerRS as indicated. (**C**) m^3^C modification levels of hctRNA^Ser^(GCU) transcript (black filled circles) and hctRNA^Ser^(GCU)-C32G (blue filled squares) by METTL6-SerRS. (**D**) m^3^C modification levels of hctRNA^Ser^(GCU) (black filled circles), hctRNA^Thr^(CGU) (blue filled squares) and hctRNA^Arg^(UCU) (green filled triangles) transcripts by METTL6-SerRS. (**E**) m^3^C modification levels of hctRNA^Ser^(GCU) transcript (black filled circles) and hctRNA^Ser^(GCU)-v-Thr (blue filled squares), -v-Leu (green filled triangles) and -v-Sec (orange filled inverted triangles) by METTL6-SerRS; (**F**) Schematic diagram of constructing various variable stem and loop replacement mutants and (**G**) various base pair or base substitutions based on hctRNA^Ser^(GCU). m^3^C modification levels of hctRNA^Ser^(GCU) transcript (black filled circles) and hctRNA^Ser^(GCU)-e11 (C-G) (blue filled squares), -e12 (U-G) (green filled triangles), -e13 (C-G) (orange filled inverted triangles), -e14 (U-G) (purple filled diamond) and -ΔUe3 (red hollow circle) (**H**); of hctRNA^Ser^(GCU) transcript (black filled circles) and hctRNA^Ser^(GCU)-Ue1G (blue filled squares), -Ce2A (green filled triangles), -Ue3G (orange filled inverted triangles), -e12 (A-U) (purple filled diamond) and -e12 (G-C) (red hollow circle) (**I**); of hctRNA^Thr^(CGU) transcript (black filled circles) and hctRNA^Thr^(CGU)-v-Ser (blue filled squares) (**J**) by METTL6-SerRS. Data represent averages of two independent experiments except three independent experiments (C) and the corresponding standard deviation.

Subsequently, we overexpressed and purified METTL6 from *E. coli* (Supplementary Figure S7C). We initially incubated METTL6 with t^6^A-modified hctRNA^Ser^(GCU) based on the above data showing that METTL2A requires t^6^A37 for efficient m^3^C32 methylation. However, no methylation product was observed (Figure 5A), indicating that purified METTL6 alone is inactive or is active but requires other cofactors for modification. Considering that yeast SerRS stimulates the activity of *Sc*Trm140 (9) and that METTL6 interacts with SerRS (Supplementary Figure S7B), we further purified human cytoplasmic SerRS (encoded by *SARS1*) (Supplementary Figure S7C). The inclusion of increasing concentrations of SerRS in the activity assay reaction of METTL6 (SerRS/METTL6 ranging from 1:1 to 5:1) triggered robust m^3^C modification; however, further elevation of SerRS from 5:1 to 10:1 decreased the m^3^C modification activity of METTL6 (Figure 5A). Thus, all subsequent activity determination of METTL6 was performed with SerRS at a 5:1 ratio (SerRS/METTL6). These results clearly showed that the m^3^C modification activity of METTL6 for tRNA, at least for hctRNA^Ser^(GCU), requires the presence of SerRS. To explore whether aminoacylation or tRNA binding capacity of SerRS contributes to m^3^C32 modification of hctRNA^Ser^(GCU), we mutated Arg^317^, which is absolutely conserved in SerRSs from three domains of life and directly interacts with γ-phosphate of AMPPNP in human SerRS-AMPPNP structure (PDB No. 4RQE) but not tRNA (Supplementary Figure S8A) (31), to Ala. SerRS-R317A exhibited an abolished aminoacylation activity (Supplementary Figure S8B); however, it stimulated m^3^C32 activity of METTL6 to comparable levels (Supplementary Figure S8C), indicating that tRNA binding but not aminoacylation by SerRS contributed to m^3^C32 modification by METTL6. Subsequently, to understand whether t^6^A37 is a determinant in METTL6-catalyzed methylation, we used hctRNA^Ser^(GCU) transcript as a substrate. Again, METTL6 showed no activity in the absence of SerRS (Figure 5B). In contrast to the modification of t^6^A-hctRNA^Thr^(CGU) by METTL2A, the modification of the hctRNA^Ser^(GCU) transcript was as robust as that of t^6^A-modified hctRNA^Ser^(GCU), with similar trends concerning the relative ratio of SerRS/METTL6 (5:1 ratio with the highest efficiency) (Figure 5B). These results highlighted that the t^6^A37 moiety was nonessential and contributed little to m^3^C biogenesis in hctRNA^Ser^(GCU) by METTL6.

Both positions 32 and 47d (e2) of hctRNA^Ser^(GCU) contain m^3^C modification. We then determined which position was m^3^C-modified by METTL6-SerRS. To this end, we prepared a hctRNA^Ser^(GCU)-C32G mutant, which was subsequently found to be hypomodified, suggesting that METTL6-SerRS is responsible for m^3^C32 but not m^3^C47d biogenesis (Figure 5C).

### Rationale of the lack of modification of tRNA^Thr^ by METTL6-SerRS due to the lack of the variable region of tRNA^Ser^ as a modification determinant

Subsequently, we found no modification of hctRNA^Thr^(CGU) and hctRNA^Arg^(UCU) transcripts by METTL6-SerRS (Figure 5D), suggesting that METTL6-SerRS has high specificity for only tRNA^Ser^.

With a typical long variable arm, hctRNA^Ser^, together with hctRNA^Leu^ and hctRNA^Sec^, constitute all the class II tRNAs in human cells. To determine any role of the long variable arm, hctRNA^Ser^-v-Thr (Figure 4A) was used as a substrate in the METTL6-SerRS modification assay (Figure 5E). The results showed that the replacement of the variable arm abolished m^3^C32, indicating a critical role of the long variable arm. Furthermore, the variable arm of hctRNA^Ser^(GCU) was changed to that of class II hctRNA^Sec^ or hctRNA^Leu^(UAG) (Figure 5F). Similarly, the data showed that neither hctRNA^Ser^(GCU)-v-Sec nor hctRNA^Ser^(GCU)-v-Leu was m^3^C32-modified by METTL6-SerRS (Figure 5E). The above data collectively revealed that METTL6-SerRS modified hctRNA^Ser^(GCU) depending on the sequence of the long variable arm.

Subsequently, we performed a sequence comparison among the variable arms of hctRNA^Ser^(GCU), hctRNA^Sec^ and hctRNA^Leu^(UAG). The variable arm of hctRNA^Ser^(GCU) most resembles that of hctRNA^Leu^(UAG) (Figure 5F), sharing U44, Ue1, Ce2, G47 and C48, but with a reduced size of the variable loop and a different variable stem. Targeting the different base pairs, we initially replaced each base of hctRNA^Ser^(GCU) with that of hctRNA^Leu^(UAG), including e11 (C–G), e12 (U-G), e13 (C-G) and e14 (U-G). Additionally, to obtain different loop sizes, we deleted Ue3 (ΔUe3) (Figure 5G). Methylation determination using the above hctRNA^Ser^(GCU) mutants showed that, in comparison to wild-type hctRNA^Ser^(GCU), the modification of hctRNA^Ser^(GCU)-ΔUe3 and -e12 (U–G) was completely abolished; that of hctRNA^Ser^(GCU)-e11 (C-G) was dramatically reduced, while that of hctRNA^Ser^(GCU)-e13 (C–G) or -e14 (U–G) was not affected at all (Figure 5H). These data suggested that the e12 base pair and loop size and/or sequence were critical for m^3^C32 modification. We further replaced e12 (U-A) with e12 (A–U) or e12 (G–C) and constructed hctRNA^Ser^(GCU)-Ue1G, -Ce2A and -Ue3G (Figure 5G). We found that a single point mutation at position Ue1 or Ce2 decreased m^3^C32 modification and that mutation at Ue3 abolished methylation, while the modification of hctRNA^Ser^(GCU)-e12 (A–U) or -e12 (G–C) decreased to different extents (Figure 5I). It is worth noting that the Ce2A mutant from Ce2 (namely, C47d) could still be m^3^C-modified, again confirming that METTL6 introduces methylation at position C32 but not Ce2 (C47d).

In addition, in contrast to hctRNA^Thr^(CGU), after transplanting the variable arm of tRNA^Ser^, hctRNA^Thr^(CGU)-v-Ser (Figure 4A) clearly gained m^3^C32 modification by METTL6-SerRS, although with clearly lower efficiency than hctRNA^Ser^(GCU) (Figure 5J).

Altogether, these results revealed that hctRNA^Thr^ was not a substrate of METTL6-SerRS due to lacking the long variable arm of hctRNA^Ser^; the base pairs e11 and e12 and residues e1 and e3 are among the critical nucleotide elements for the m^3^C32 modification of hctRNA^Ser^(GCU) by METTL6-SerRS.

### Anticodon loop harbors key determinants for m^3^C32 modification by METTL6-SerRS

**Figure 6.**
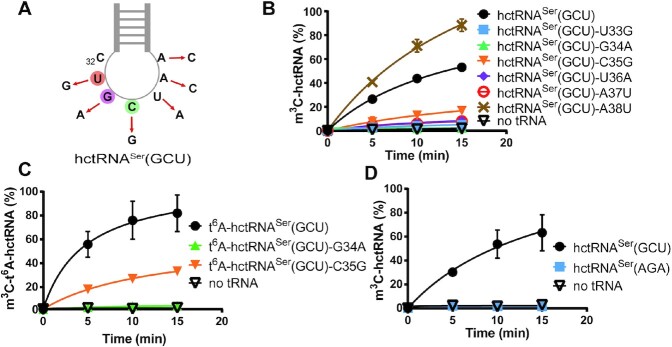
The anticodon loop critically determines m^3^C32 formation in hctRNA^Ser^ (GCU) by METTL6-SerRS. (**A**) Schematic diagram showing the construction of six hctRNA^Ser^(GCU) mutants. m^3^C modification levels of hctRNA^Ser^(GCU) transcript (black filled circles) and hctRNA^Ser^(GCU)-U33G (blue filled squares), -G34A (green filled triangles), -C35G (orange filled inverted triangles), -U36A (purple filled diamonds), -A37C (red hollow circles) and -A38C (brown forks) (**B**); of t^6^A-hctRNA^Ser^(GCU) (black filled circles) and t^6^A-hctRNA^Ser^(GCU)-G34A (green filled triangles) and -C35G (orange filled inverted triangles) (**C**); of hctRNA^Ser^(GCU) transcript (black filled circles) and hctRNA^Ser^(AGA) transcript (blue filled squares) (**D**) by METTL6-SerRS. Data represent averages of three independent experiments (B) or two independent experiments (C, D) and the corresponding standard deviation.

METTL6 requires SerRS for m^3^C32 modification (Figure 5A, B) but does not directly bind it (Supplementary Figure S7B). It is well established that the long variable arm of tRNA^Ser^ is bound and recognized by SerRS (32). In addition, G35 is a critical determinant of methylation by METTL2A. All this evidence prompted us to explore whether the anticodon loop of hctRNA^Ser^(GCU) plays a potentially important role in tRNA recognition by METTL6. To this end, we changed each base of the anticodon loop of hctRNA^Ser^(GCU), except the C32 modification site (Figure 6A), resulting in the hctRNA^Ser^(GCU)-U33G, -G34A, -C35G, -U36A, -A37C, and -A38C mutants. Methylation determination using the above mutants showed that, in comparison to that of wild-type hctRNA^Ser^(GCU), modification of hctRNA^Ser^(GCU)-U33G, -G34A, -U36A and -A37C modification was completely abolished, and hctRNA^Ser^(GCU)-C35G modification was dramatically reduced, while that of hctRNA^Ser^(GCU)-A38C was unexpectedly significantly increased (Figure 6B). Among the above single-point mutants, only hctRNA^Ser^(GCU)-G34A and -C35G exhibited comparable levels of t^6^A modification by Sua5/KEOPS; therefore, we prepared t^6^A-modified hctRNA^Ser^(GCU)-G34A and -C35G. The results showed that t^6^A-hctRNA^Ser^(GCU)-G34A was still hypomodified, while the modification efficiency for t^6^A-hctRNA^Ser^(GCU)-C35G was slightly elevated compared with its transcript (Figure 6B, C), suggesting that the presence of t^6^A37 compensates for the loss of the optimal anticodon sequence.

hctRNA^Ser^(AGA) also harbors m^3^C32 modification *in vivo* (5); however, it has a different anticodon from that of hctRNA^Ser^(GCU). Subsequent modification assays showed that, in contrast to hctRNA^Ser^(GCU), hctRNA^Ser^(AGA) transcript was unable to be modified by METTL6-SerRS (Figure 6D). This result, together with the stimulatory role of t^6^A37 in the hctRNA^Ser^(GCU)-C35G mutant, suggested that the presence of i^6^A37 modification in hctRNA^Ser^(AGA) is likely a key determinant of its m^3^C32 modification.

In summary, in addition to the long variable arm, the anticodon loop of hctRNA^Ser^(GCU) contains key elements that determine m^3^C32 biogenesis by METTL6-SerRS.

### The NTD and CTD domains of METTL2A and METTL6 are mutually incompatible

METTL2A and METTL6 share highly similar CTD domains. However, the NTD domain of METTL6 is sharply truncated or degenerated compared with that of METTL2A. Which domain or element in enzymes determines their totally distinct substrate specificity is unclear. Therefore, we switched the corresponding NTD domains to understand whether the substrate preference could be artificially altered. We fused the NTD of METTL6 with the CTD of METTL2A to obtain N6-METTL2A; similarly, N2-METTL6 was also constructed (Supplementary Figure S9A).

We purified both N6-METTL2A and N2-METTL6 from *E. coli*. *In vitro* methylation assays showed that neither enzyme was able to introduce methylation at t^6^A-hctRNA^Thr^(CGU) (Supplementary Figure S9B), suggesting that both the METTL2A NTD and CTD domains are critical for its m^3^C32 modification activity. In the presence of SerRS, we also determined the modification of t^6^A37-hypomodified or t^6^A37-modified hctRNA^Ser^(GCU) by the two chimeric enzymes. Similarly, no modification was observed (Supplementary Figure S9C, D). In addition, considering that METTL2A requires G35 as a positive determinant, we also modified t^6^A-hctRNA^Ser^(GCU)-C35G; again, the two chimeric enzymes generated no m^3^C32 (Supplementary Figure S9E).

Furthermore, we expressed the genes encoding METTL2A, METTL6, N6-METTL2A and N2-METTL6 in HEK293T cells. However, the two chimeric mutants were not detected in the WCL, probably due to inefficient expression or rapid degradation after synthesis (Supplementary Figure S9F).

Above all, these data collectively suggested that the NTD and CTD domains of METTL2A and METTL6 were mutually incompatible in expression/stability *in vivo* and for m^3^C32 modification *in vitro*.

## DISCUSSION

Only a single Mettl2 is present in some eukaryotes, such as mice; however, the simultaneous existence of two nearly identical m^3^C32 methyltransferases with the same cellular distribution, METTL2A and METTL2B, in others, such as human, is puzzling (8). We showed here that the activity of METTL2B is far lower than that of METTL2A. Notably, among the three different variations between the two enzymes, alternative rigid Pro residue is frequently observed. Indeed, the P124C mutation alone in METTL2A is sufficient to dramatically reduce its activity to levels comparable to those of METTL2B. Considering that DALRD3 is required for m^3^C32 formation in tRNA^Arg^(CCU) and tRNA^Arg^(UCU) isoacceptors in human cells (11), it is likely that these amino acid variations between two enzymes, especially the presence or absence of rigid Pro, frequently function in determining local/global protein conformation and fine-tune protein conformation and/or structure, which may influence their interaction with DARLD3 to control the tRNA^Arg^ methylation level. Alternatively, METTL2B possibly needs other unknown co-factors in catalyzing tRNA modification. On the other hand, tRNA modification has been shown to be highly dynamic in response to various stimuli or stresses (33,34). It is possible that the m^3^C32 modification level is precisely balanced based on the expression level or ratio of METTL2A and METTL2B, which is regulated in a tissue- or cell-specific manner.

The deficiency of METTL2A in modifying tRNA^Arg^ is intriguing, considering that tRNA^Arg^ and tRNA^Thr^, both class I tRNAs, have similar secondary and tertiary structures, in contrast to class II tRNA^Ser^. The presence of a tRNA binding motif in DALRD3 implies that METTL2A is inefficient in binding tRNA^Arg^ and is probably assisted by DALRD3. However, the exact functional assignment of METTL2A-DALRD3 remains unclear.

We also observed that once G35 is introduced into hctRNA^Ser^(GCU), in the presence of t^6^A37, the tRNA mutant gains the ability to be modified by METTL2A, although further deletion of the long variable arm significantly elevates the modification level. However, in human cells, most hctRNA^Ser^ species, including hctRNA^Ser^(AGA), hctRNA^Ser^(UGA) and hctRNA^Ser^(CGA), naturally contain a G35. Sure, in these hctRNA^Ser^ species with G35, i^6^A37 is present instead of t^6^A37. In addition to the long variable arm, whether C35 and t^6^A37 in hctRNA^Ser^(GCU) or G35 and i^6^A37 in other hctRNA^Ser^ species synergistically determine substrate specificity by METTL6-SerRS but not METTL2A is unknown and needs further exploration.

One remarkable difference revealed here is that METTL6 does not require modification at residue 37 as a prerequisite, at least in modification for hctRNA^Ser^(GCU). On the other hand, m^3^C32 formation by METTL6 is dependent on the presence of SerRS. METTL6 did not directly interact with SerRS. Increasing ratio of SerRS to METTL6 from 5:1 to 10:1 decreased methylation activity of METTL6; we suggested that the affinity between components of METTL6-tRNA^Ser^-SerRS ternary complex is not strong and too much SerRS might form SerRS-tRNA^Ser^ binary complex and thus competes with formation of ternary complex. On the other hand, the possibility of presence of trace amount of nuclease in the SerRS sample cannot be absolutely excluded. Perplexingly, a recent report shows that purified GST-tagged METTL6 alone is able to introduce m^3^C32 modification into total cellular RNA (35). hctRNA^Ser^(GCU) transcript was used in this work. Although the possibility that other modifications in tRNA^Ser^ species eliminate the requirement of SerRS cannot be absolutely excluded, we suggest that the modification of total RNA may not be derived from tRNA modification. Alternatively, modifications other than hctRNA^Ser^(GCU) used here, including hctRNA^Ser^(AGA), hctRNA^Ser^(UGA) or hctRNA^Ser^(CGA), could be performed by METTL6 alone. However, when the hctRNA^Ser^(UGA) transcript was used in an *in vitro* methylation assay, only a basal level of modification (with CPM 100–300) was observed (35). Therefore, we hypothesize that the m^3^C32 activity of METTL6 for hctRNA^Ser^ is critically dependent on SerRS. The biological function of m^3^C32 modification, despite being currently unidentified, should be of high significance considering that multiple tRNA methyltransferases and cofactors have evolved in human cells. Thus, SerRS is indeed a key multifaceted regulator in protein synthesis, vascular development and other functions, such as tRNA modification (36).

m^3^C modification has been shown to be present in mRNA (8). The independence of the t^6^A modification of METTL6 elicits another interesting question: is METTL6 able to introduce m^3^C to RNA species other than tRNA? To date, t^6^A modification has been detected only in tRNAs and not found in mRNAs. Therefore, in combination with our findings that METTL2A is dependent on t^6^A modification prior to m^3^C biogenesis, METTL6 is more likely to form m^3^C in RNAs other than METTL2A. Previous work has shown that yeast Trm140 recognizes *Sc*tRNA^Thr^s and *Sc*tRNA^Ser^(GCU) depending on t^6^A37 and *Sc*tRNA^Ser^(AGA), (CGA) and (UGA) depending on i^6^A37. In addition, XGU and t^6^A37 are sufficient for m^3^C32 modification of yeast tRNA^Thr^s (9). Our work shows that the modification of tRNA^Thr^s by human METTL2A also requires an anticodon stem and that human METTL6 recognizes hctRNA^Ser^(GCU) independently of t^6^A, suggesting that humans and yeasts have different mechanisms for recognizing tRNA substrates. Indeed, cellular mRNAs frequently form anticodon stem and loop-like architectures, which are responsible for recruiting interacting protein partners, such as aminoacyl-tRNA synthetases (37). In addition, some noncoding RNAs contain tRNA-like structures (38). Although a previous report indicated no obvious change in m^3^C abundance in the mRNA fraction in Mettl6 knockout mice (8), the frequency of m^3^C modification by METTL6 may be low to be accurately captured. Indeed, a recent m^3^C mapping analysis revealed little m^3^C abundance in mRNA (20).

Mettl8 has been shown to participate mRNA m^3^C biogenesis (8). However, nothing is known about the mRNA substrate selection mechanism of Mettl8. Mettl8 resembles METTL2A most closely in primary sequence. Our clarification of the tRNA selection recognition pattern by METTL2A, especially the key role of the anticodon stem and loop region of tRNA, provides valuable insights into how METTL8 recognizes mRNA substrates. Considering that t^6^A modification has not been detected in mRNA, whether Mettl8 does not rely on t^6^A modification is an open question. Moreover, our work showed that neither METTL6 nor METTL2A was significantly localized in mitochondria, while a recent study showed that METTL8 was localized in mitochondria (39). Therefore, we reasonably infer that METTL8 is highly likely to be responsible for the m^3^C32 modification of mitochondrial tRNAs.

**Figure 7.**
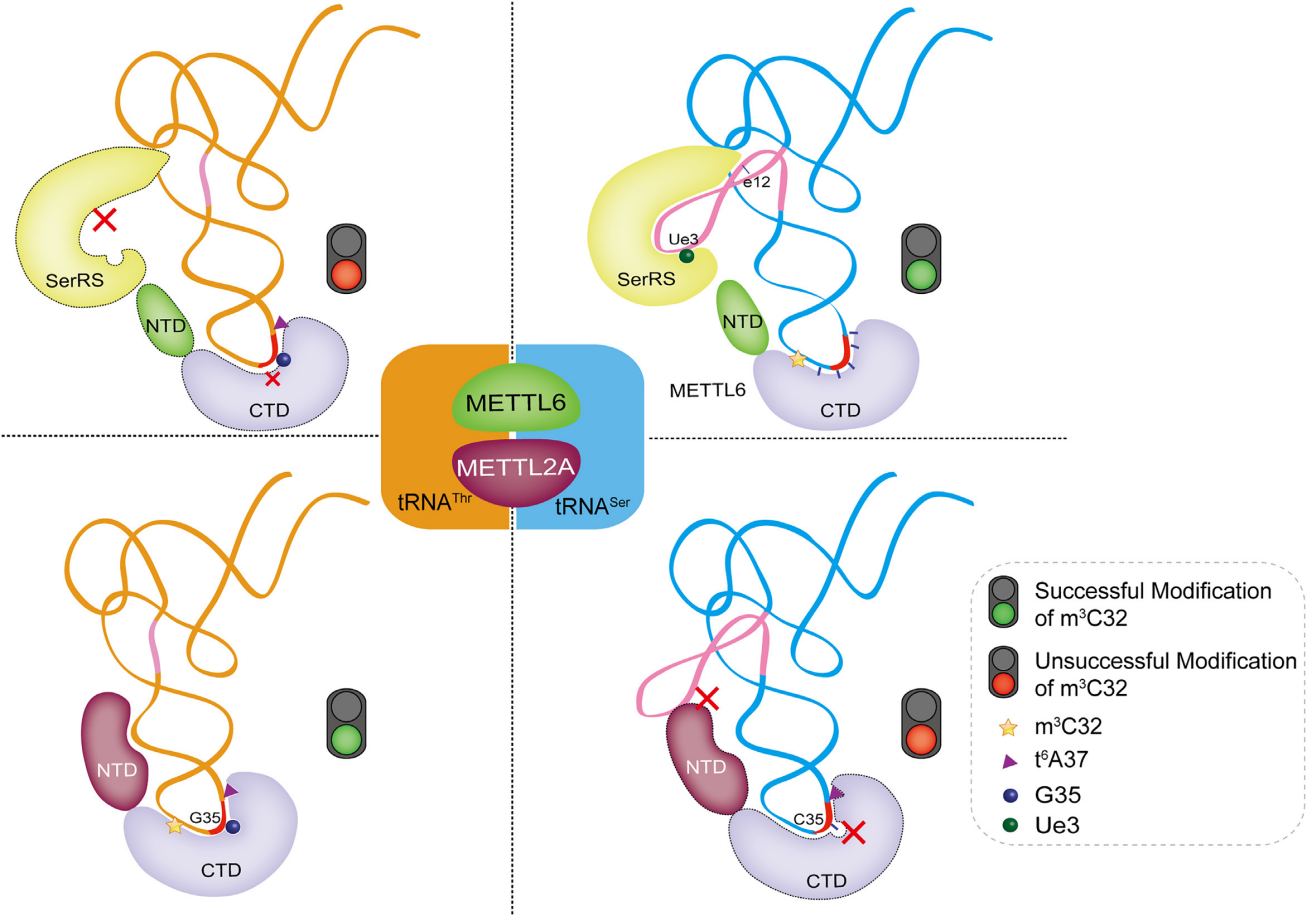
Model of mutually exclusive substrate selection strategy by human m^3^C RNA transferases METTL2A and METTL6. When hctRNA^Thr^s are modified by METTL2A, the key elements in the anticodon loop (t^6^A37 and G35) are recognized by the CTD, and other key elements, including the anticodon stem, are recognized by the long NTD (lower left quadrant). hctRNA^Ser^ (GCU) is modified by METTL6 with the assistance of SerRS, which recognizes the essential elements on the anticodon loop (bases 33–37) and variable arm (including base pair e12 and Ue3). The truncated NTD of METTL6 is unable to bind tRNA as efficiently as that of METTL2A (upper right quadrant). hctRNA^Thr^s are not modified by METTL6-SerRS due to the unfavorable anticodon loop and lack of the long variable arm (upper left quadrant); in parallel, hctRNA^Ser^(GCU) is not modified by METTL2A due to the lack of G35 and presence of a long variable arm (lower right quadrant). The variable arms are indicated in pink.

The CTDs for binding SAM are highly conserved, while the most striking sequence difference is observed in the NTDs between METTL2A and METTL6. The NTD of METTL6 is significantly truncated only with a conserved ‘FFKDR’ motif; however, that of METTL2A is a much longer domain even than those of *Sc*Trm140 and *Sp*Trm140 (Supplementary Figure S2). In combination with our revealed tRNA sequence requirement for both hctRNA^Thr^s and hctRNA^Ser^(GCU), we proposed a model for multiple substrate selection and modification by multiple m^3^C32 modification enzymes (Figure 7). The CTD of both enzymes recognizes the anticodon loop region, relying on distinct sets of determinants in hctRNA^Thr^s (G35 and t^6^A37) and hctRNA^Ser^(GCU) (positions 33–37); the long NTD of METTL2A recognizes other elements, such as the anticodon stem, of hctRNA^Thr^s. However, the degenerated NTD of METTL6 is unable to bind hctRNA^Ser^(GCU) efficiently, which is augmented by SerRS for recognizing the long variable arm (including Ue3 and e12 base pairs), but they do not interact directly. METTL2A fails to modify hctRNA^Ser^(GCU) due to the presence of C35 in the anticodon and the long variable arm, possibly leading to spatial conflict and/or electrostatic repulsion between the variable arm and the long NTD of METTL2A. On the other hand, hctRNA^Thr^s cannot be decorated by METTL6 due to an unfavorable anticodon, the lack of the long variable arm of hctRNA^Thr^s (leading to inability to be captured by METTL6-SerRS) and the truncated NTD of METTL6 (making the efficient recognition of hctRNA^Thr^s by METTL6 alone impossible). Notably, hctRNASer(AGA) transcripts cannot be modified by METTL6-SerRS, suggesting a distinct recognition pattern between t^6^A37- and i^6^A37-harboring hctRNA^Ser^ species, which should be further explored.

## Supplementary Material

gkab603_Supplemental_File
