# Supplementary material for: Mutually exclusive substrate selection strategy by human m3C RNA transferases METTL2A and METTL6
Source: Nucleic Acids Res. 2021 Jul 15;49(14):8309–23. doi: 10.1093/nar/gkab603 (PMC8373065; doi:10.1093/nar/gkab603)
Supplement: gkab603_Supplemental_File [file gkab603_supplemental_file.docx]

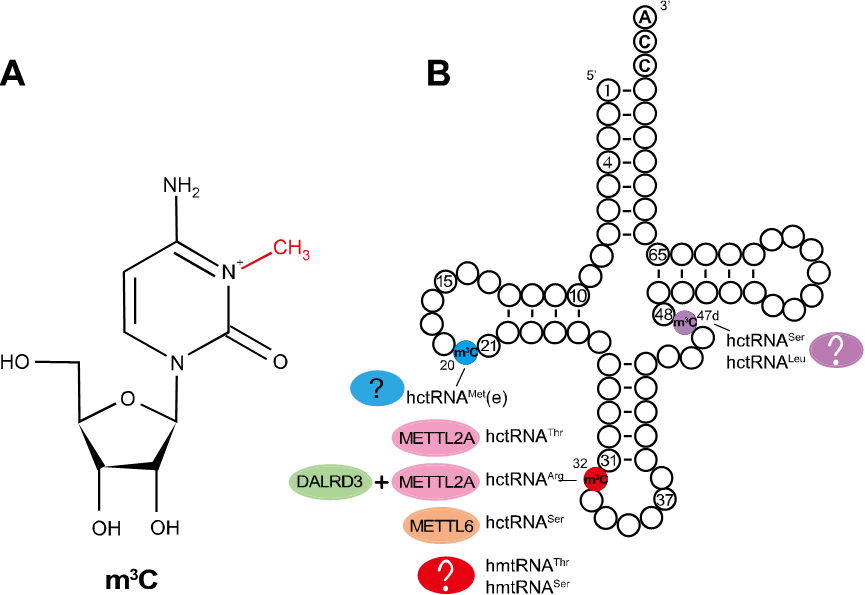


**Supplementary Figure 1. m^3^C and its modification in tRNAs.**

(**A**) Chemical structure of m^3^C. (**B**) Schematic diagram showing the position of m^3^C on tRNA and the corresponding tRNA species and enzymes responsible for these m^3^C modifications. Enzymes for modification at bases 20 and 47d and at base 32 of mitochondrial tRNAs have not been identified and are marked by question marks.

**
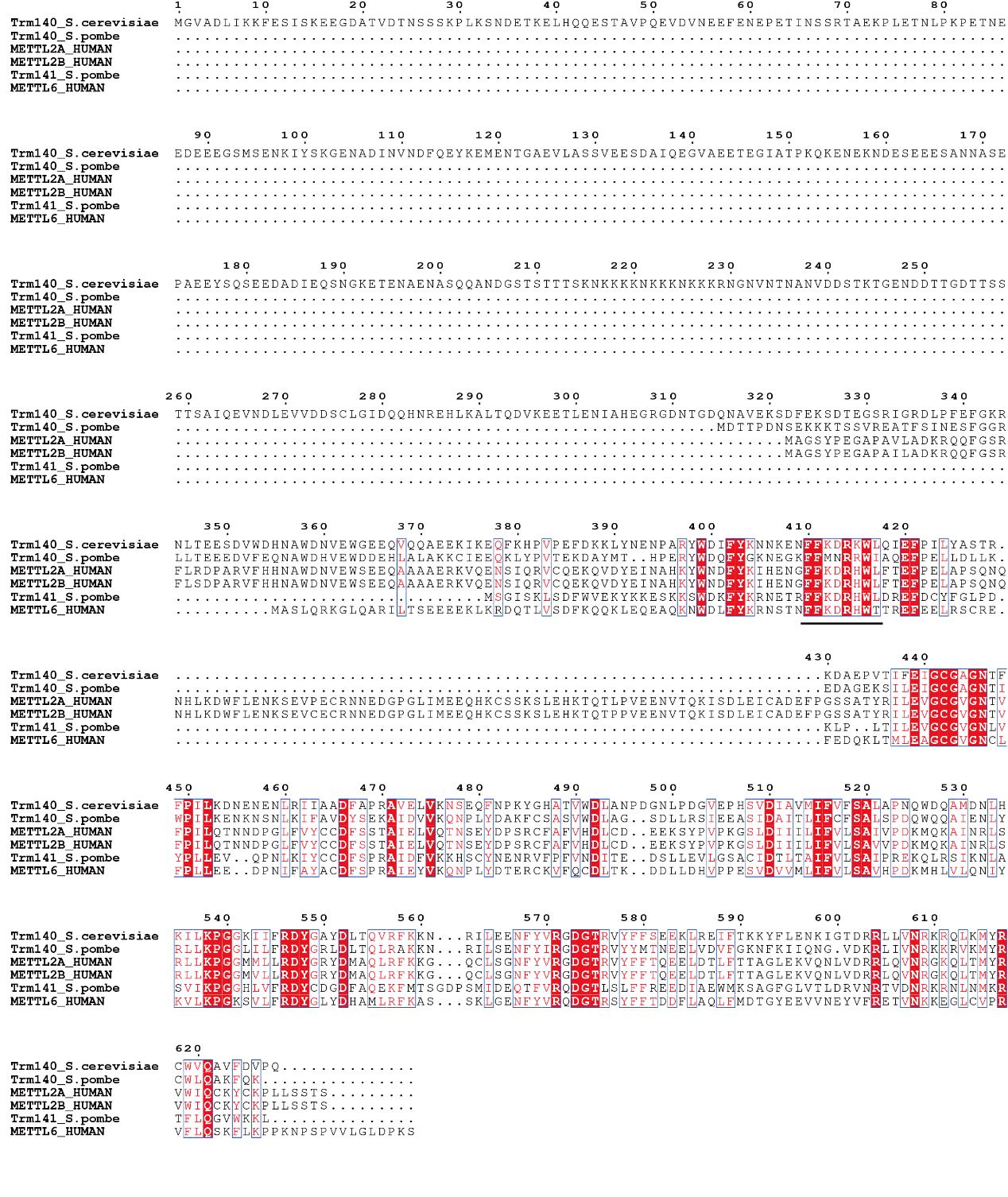
**

**Supplementary Figure 2.** **Sequence alignment of *Sc*Trm140, *Sp*Trm140, *Sp*Trm141, METTL2A, METTL2B and METTL6.**

The amino acid residues of *Sc*Trm140 are labeled on the top. The N-terminal conserved “FFKDR” motifs are indicated.


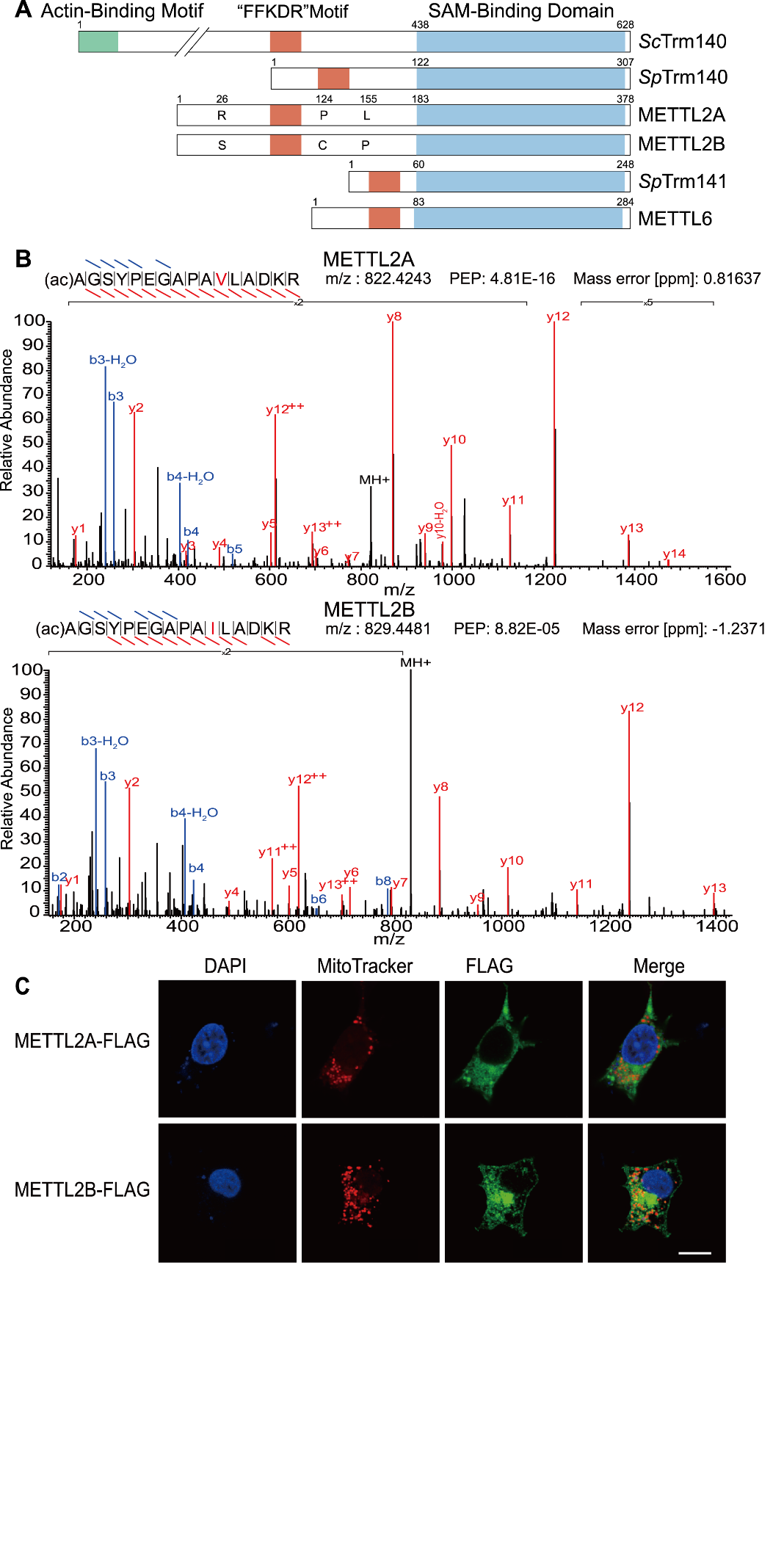


**Supplementary Figure 3. Both METTL2A and METTL2B are expressed *in vivo.***

(**A**) Schematic representation showing the domain compositions of *Sc*Trm140, *Sp*Trm140, *Sp*Trm141, METTL2A, METTL2B and METTL6 based on sequence alignment, and amino acids with completely different side chain properties in METTL2A and METTL2B are indicated. (**B**) LC-MS analysis of the WCL of HEK293T cells. (**C**) Cellular localization of overexpressed METTL2A-FLAG (upper panel) and METTL2B-FLAG (lower panel) in HEK293T cells analyzed by fluorescence microscopy. Scale bar: 10 μm.


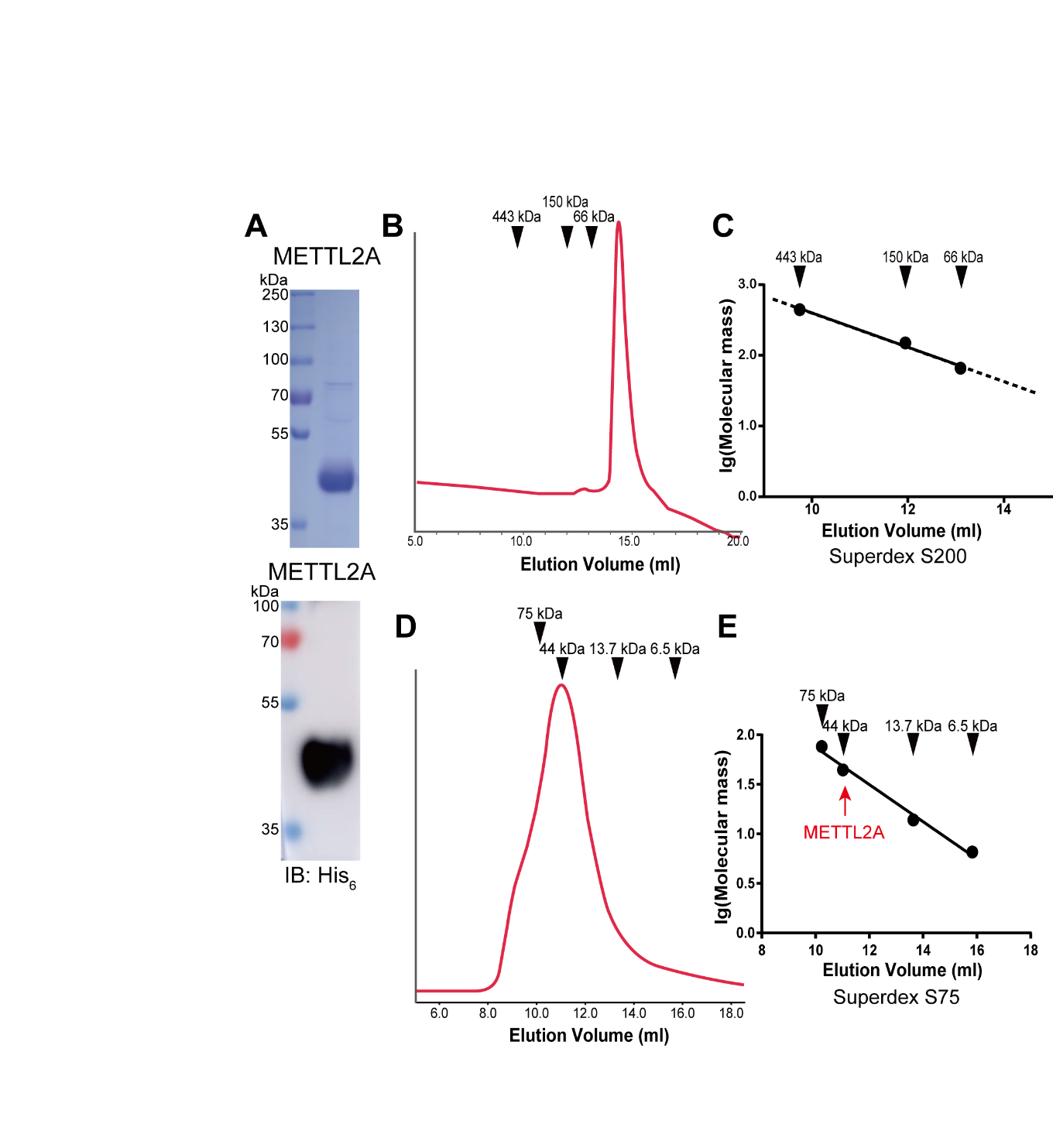


**Supplementary Figure 4.** **Purified METTL2A is a monomer.**

(**A**) SDS-PAGE analysis of purified METTL2A from *E. coli*. The protein was stained with Coomassie blue (upper panel) and confirmed by Western blot (lower panel). Gel filtration analysis of purified METTL2A using Superdex S200 (**B**) and Superdex S75 (**D**) with the elution volumes of standard proteins of known molecular mass indicated. Determination of the molecular mass of purified METTL2A based on the elution volumes of the abovementioned standard proteins using Superdex S200 (**C**) and Superdex S75 (**E**).


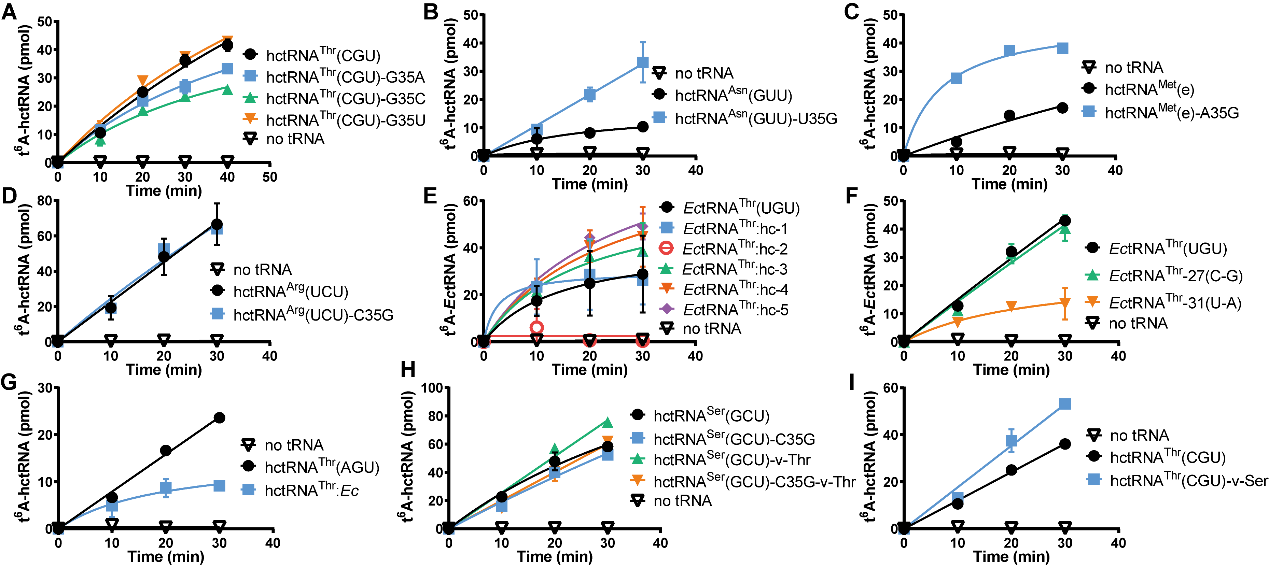


**Supplementary Figure 5. t^6^A modification levels of hctRNAs.**

t^6^A modification levels of tRNA^Thr^(CGU) and tRNA^Thr^(CGU)-G35A, -G35C and -G35U (**A**); of tRNA^Asn^(GUU) and tRNA^Asn^(GUU)-U35G (**B**); of tRNA^Met^(e) and tRNA^Met^(e)-A35G (**C**); of tRNA^Arg^(UCU) and tRNA^Arg^(UCU)-C35G (**D**); of *Ec*tRNA^Thr^(UGU) and mutants *Ec*tRNA^Thr^:hc-1-5 replaced with tRNA^Thr^(AGU) (**E**); of *Ec*tRNA^Thr^(UGU) and mutants *Ec*tRNA^Thr^-27(C-G) and -32(U-A) (**F**); of hctRNA^Thr^(AGU) and mutants hctRNA^Thr^:*Ec* (**G**); of tRNA^Ser^(CGU) and tRNA^Ser^(CGU)-C35G, -v-Thr and -C35G-v-Thr (**H**) and of hctRNA^Thr^(CGU) and mutants hctRNA^Thr^(CGU)-v-Ser (**I**) by Sua5/KEOPS. Data represent averages of two independent experiments and the corresponding standard deviation.


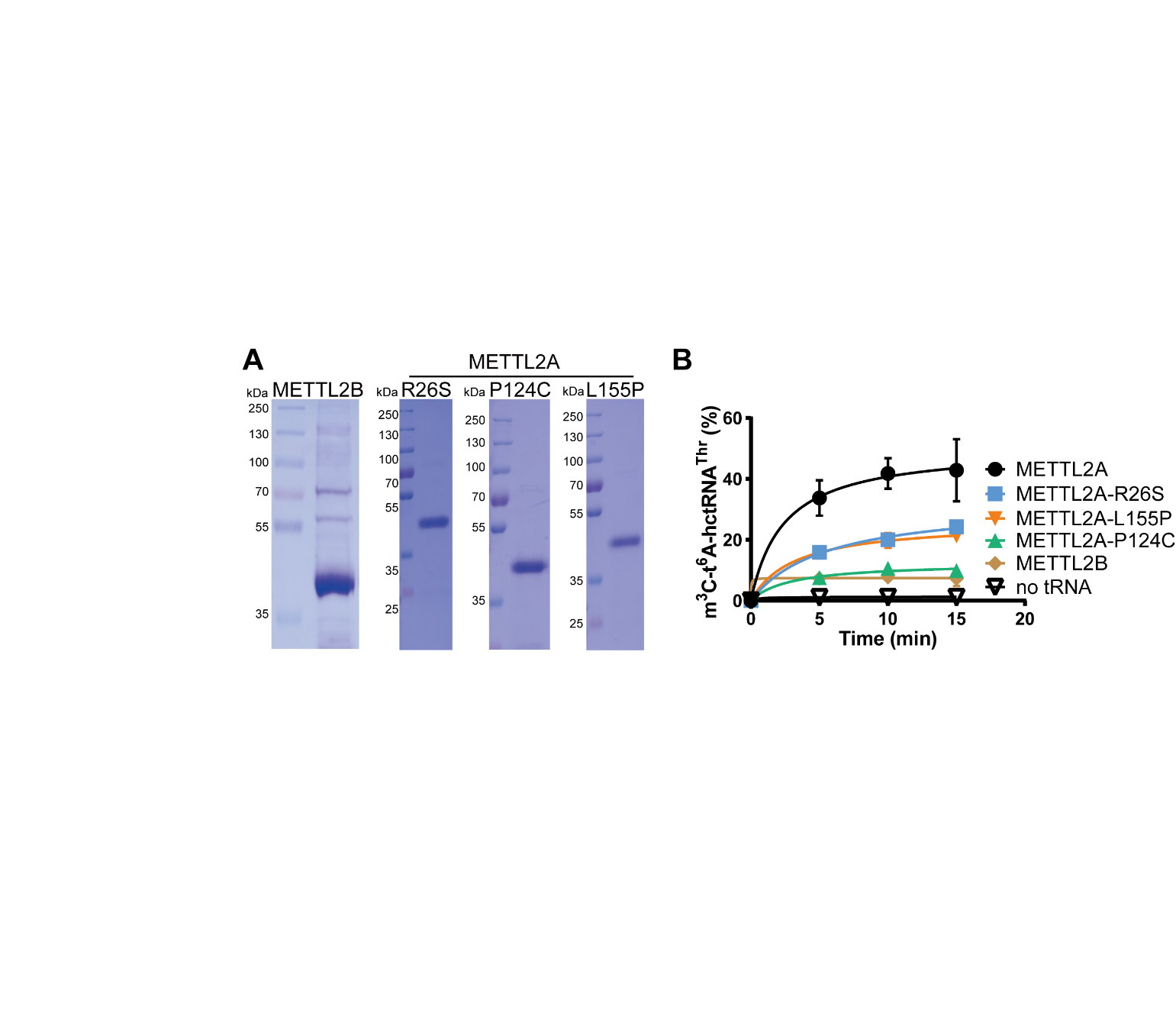


**Supplementary** **Figure 6.** **METTL2B exhibited little m^3^C32 modification activity *in vitro.***

(**A**) SDS-PAGE analysis of purified METTL2B and METTL2A-R26S, -P124C and -L155P (from left to right) from *E. coli*. The proteins were stained with Coomassie blue. (**B**) m^3^C32 modification levels of t^6^A-hctRNA^Thr^(CGU) by METTL2B (brown filled diamond), METTL2A (black filled circles) and its mutants -R26S (blue filled squares), -P124C (green filled triangles) and -L155P (orange filled inverted triangles). Data represent averages of three independent experiments and the corresponding standard deviation.


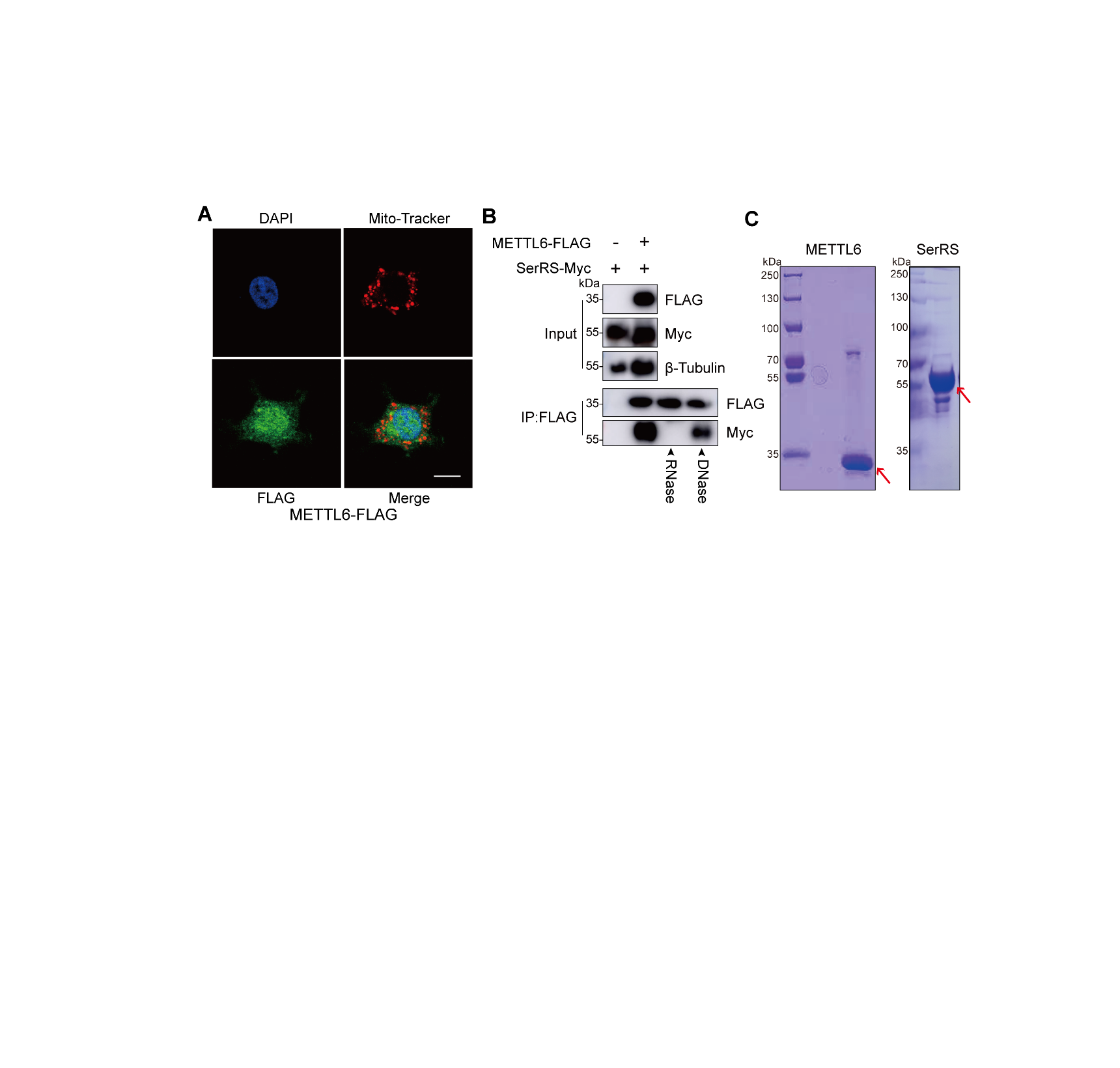


**Supplementary** **Figure 7. METTL6 interacts with SerRS in an RNA-dependent mode.**

(**A**) Cellular localization of overexpressed METTL6-FLAG in HEK293T cells analyzed by immunofluorescence. Scale bar: 10 μm. (**B**) Genes encoding METTL6-FLAG and SerRS-Myc were coexpressed in HEK293T cells. SerRS-Myc was precipitated by METTL6-FLAG using anti-FLAG antibodies in the presence of DNase I but not RNase A treatment. (**C**) SDS-PAGE analysis of purified METTL6 and SerRS from *E. coli*. The protein was stained with Coomassie blue.


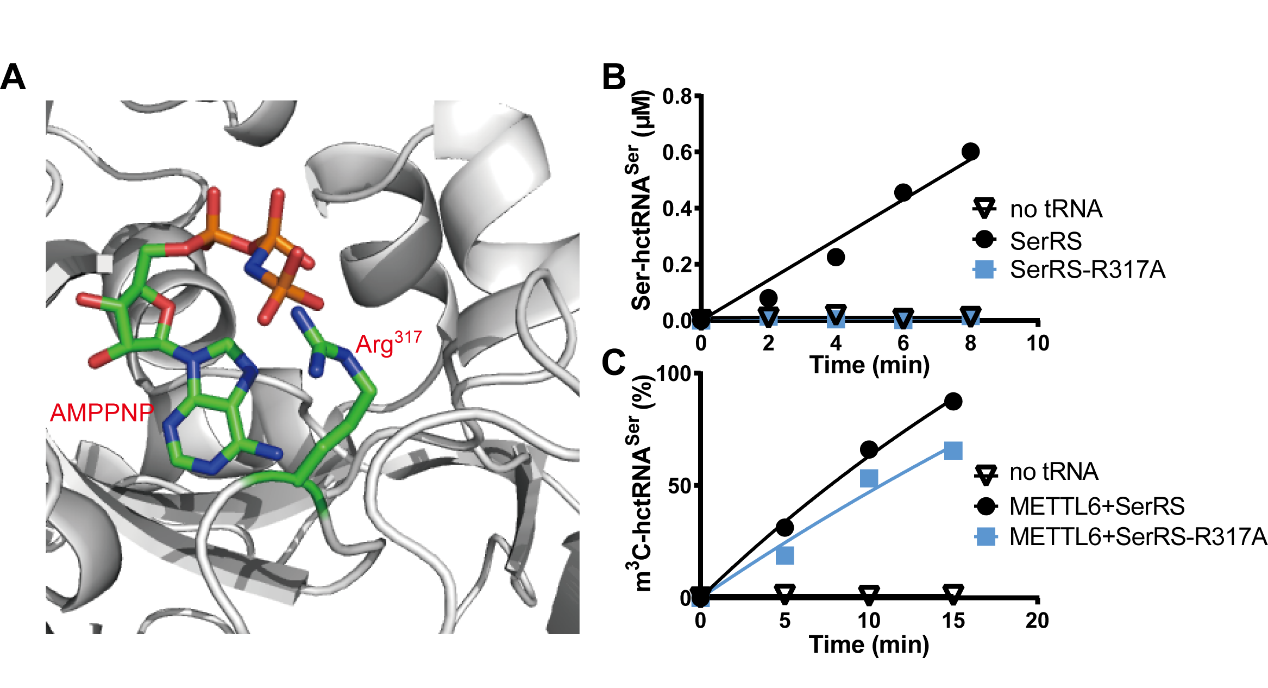


**Supplementary Figure 8. Aminoacylation of SerRS is not required for m^3^C32 by METTL6.**

(**A**) Structure of human SerRS-AMPPNP (PDB No. 4RQE) showing the direct interaction between the side chain of Arg^317^ with the γ-phosphate of AMPPNP. (**B**) Aminoacylation activities of SerRS (black filled circles) and its mutant SerRS-R317A (blue filled squares). (**C**) m^3^C32 modification levels of hctRNA^Ser^(GCU) by METTL6 with SerRS (black filled circles) and SerRS-R317A (blue filled squares). Data represent averages of two independent experiments and the corresponding standard deviation.


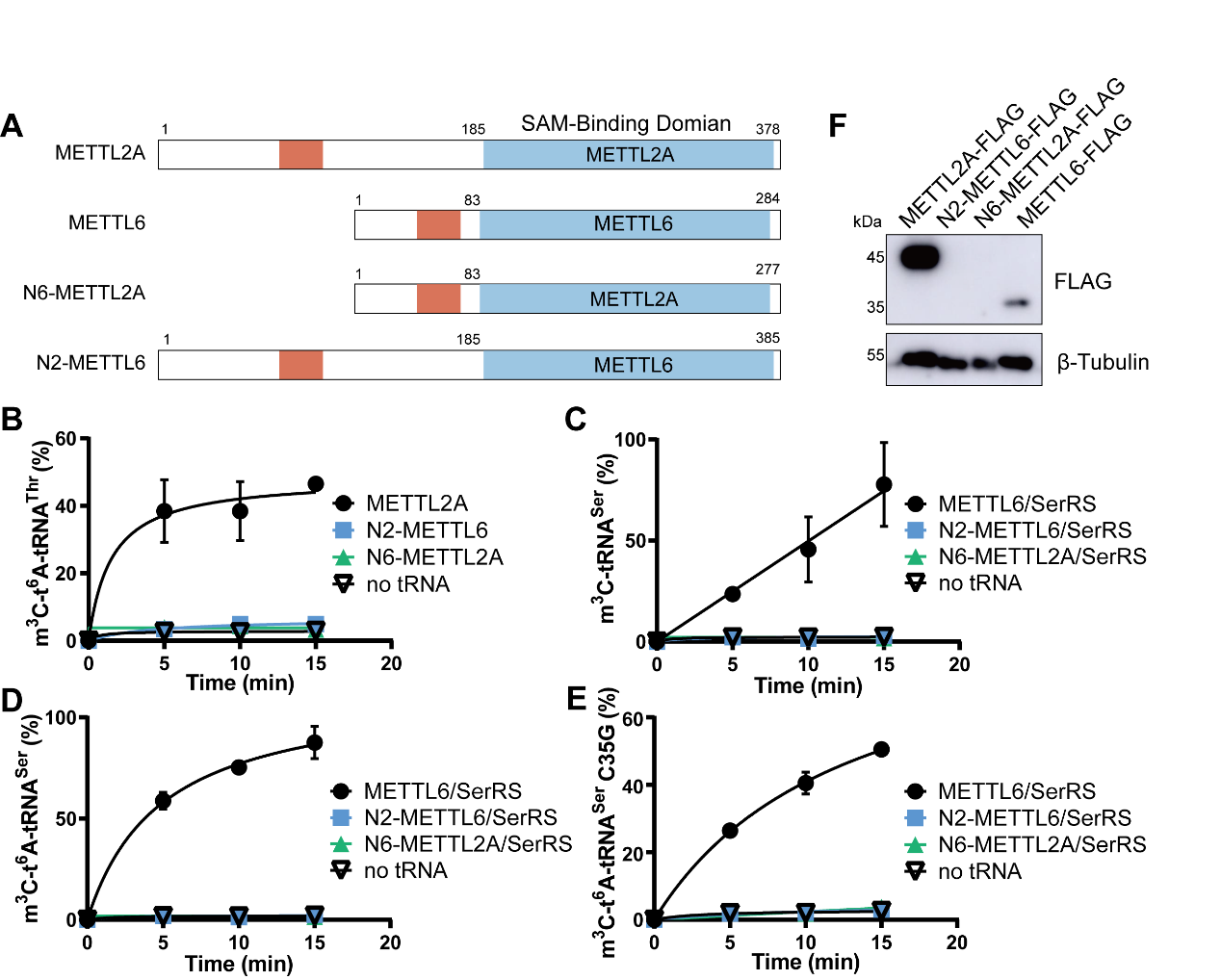


**Supplementary Figure 9. NTDs and CTDs of METTL2A and METTL6 were functionally incompatible.**

(**A**) Schematic representation showing the domain compositions of METTL2A and METTL6 and their mutants after switching the NTDs. (**B**) m^3^C modification levels of t^6^A-hctRNA^Thr^ (CGU) by METTL2A (black filled circles), N2-METTL6 (blue filled squares) and N6-METTL2A (orange filled triangles). m^3^C modification levels of hctRNA^Ser^(GCU) transcript (**C**), t^6^A-hctRNA^Ser^(GCU) (**D**) and t^6^A-tRNA^Ser^(GCU)-C35G (**E**) by METTL6-SerRS (black filled circles), N2-METTL6-SerRS (blue filled squares) and N6-METTL2A-SerRS (orange filled triangles). (**F**) Protein levels of METTL2A, METTL6, N6-METTL2A and N2-METTL6 in HEK293T cells. Data represent averages of two independent experiments and the corresponding standard deviation.

**Supplementary Table 1. m^3^C32 methyltransferases of different species**

| **Species** | **Substrate** | **Methyltransferases** |
| --- | --- | --- |
| *S. cerevisiae* | *Sc*tRNA^Thr^  *Sc*tRNA^Ser^ | Trm140 |
| *S. pombe* | *Sp*tRNA^Thr^ | Trm140 |
|  | *Sp*tRNA^Ser^ | Trm141 |
| *M. musculus* | mctRNA^Thr^ | Mettl2 |
|  | mctRNA^Arg^ |  |
|  | mctRNA^Ser^ | Mettl6 |
| *H. sapiens* | hctRNA^Thr^ | METTL2A/B |
|  | hctRNA^Arg^ | METTL2A/B+DALRD3 |
|  | hctRNA^Ser^ | METTL6 |
|  | hmtRNA^Thr^ | ? |
|  | hmtRNA^Ser^ |  |

**Supplementary Table 2. Primers**

| Name | Primer (5’ to 3’) (restriction sites italic) |
| --- | --- |
| METTL2A-F | gc*GAGCTC*atggccggctcctaccct |
| METTL2A-R | gc*GCGGCCGC*tcagctggtgctggacag |
| METTL6-F | gc*GAGCTC*atggcttctttgcaaaggaaag |
| METTL6-R | gc*GCGGCCGC*tcaggacttaggatccag |
| SerRS-F | gc*CATATG*atggtgctggatctggatttg |
| SerRS-R | gc*CTCGAG*tcaagcatcggtgacctcc |
| pTrc99b-T7-F | atgtgtggaattgtgagcgg |
| hctRNA^Thr^(AGU)-R | tggaggcaccgctggg |
| hctRNA^Thr^(CGU)-R | tggaggcacggacggg |
| hctRNA^Thr^(UGU)-R | tggaggccccagcgag |
| hctRNA^Ser^(GCU)-R | tggcgacgagggtggg |
| hctRNA^Arg^(CCU)-R | tggtaccccaggtggg |
| hctRNA^Arg^(UCU)-R | tggatctctgccggg |
| hctRNA^Asn^(GUU)-R | tggcgtccctgggtgg |
| hctRNA^Met^(e)-R | tggtgccctctctgag |
| *Ec*tRNA^Thr^(UGU)-R | tggtgccgactaccgg |
